# Neuronal Growth Cone Size-Dependent and -Independent Parameters of Microtubule Polymerization

**DOI:** 10.3389/fncel.2018.00195

**Published:** 2018-07-17

**Authors:** Alexa Kiss, Irmgard Fischer, Tatjana Kleele, Thomas Misgeld, Friedrich Propst

**Affiliations:** ^1^Department of Biochemistry and Cell Biology, Max F. Perutz Laboratories, University of Vienna, Vienna Biocenter, Vienna, Austria; ^2^Institute of Neuronal Cell Biology, Technical University of Munich, Munich Cluster for Systems Neurology (SyNergy) and German Center for Neurodegenerative Diseases (DZNE), Munich, Germany

**Keywords:** dorsal root ganglia, quantitative microscopy, microtubule-associated proteins, MAP1B, EB3, multiscale analysis, image processing, growth cone

## Abstract

Migration and pathfinding of neuronal growth cones during neurite extension is critically dependent on dynamic microtubules. In this study we sought to determine, which aspects of microtubule polymerization relate to growth cone morphology and migratory characteristics. We conducted a multiscale quantitative microscopy analysis using automated tracking of microtubule plus ends in migrating growth cones of cultured murine dorsal root ganglion (DRG) neurons. Notably, this comprehensive analysis failed to identify any changes in microtubule polymerization parameters that were specifically associated with spontaneous extension vs. retraction of growth cones. This suggests that microtubule dynamicity is a basic mechanism that does not determine the polarity of growth cone response but can be exploited to accommodate diverse growth cone behaviors. At the same time, we found a correlation between growth cone size and basic parameters of microtubule polymerization including the density of growing microtubule plus ends and rate and duration of microtubule growth. A similar correlation was observed in growth cones of neurons lacking the microtubule-associated protein MAP1B. However, MAP1B-null growth cones, which are deficient in growth cone migration and steering, displayed an overall reduction in microtubule dynamicity. Our results highlight the importance of taking growth cone size into account when evaluating the influence on growth cone microtubule dynamics of different substrata, guidance factors or genetic manipulations which all can change growth cone morphology and size. The type of large scale multiparametric analysis performed here can help to separate direct effects that these perturbations might have on microtubule dynamics from indirect effects resulting from perturbation-induced changes in growth cone size.

## Introduction

Continuous remodeling of the microtubule cytoskeleton has long been recognized as essential for neurite outgrowth and growth cone guidance in response to diverse attractive and chemo-repulsive guidance cues (Letourneau and Ressler, 1984; Challacombe et al., 1997; Kalil and Dent, 2005; Lowery and Van Vactor, 2009; Dent et al., 2011). Measuring microtubule dynamics in mammalian neurons via fluorescently labeled tubulin proved to be challenging due to the high density of microtubules in neurites and the comparatively small size of their growth cones. With the discovery of proteins that bind specifically to plus ends of polymerizing microtubules such as CLIP-170 (Perez et al., 1999) and end-binding (EB) protein 1 (Mimori-Kiyosue et al., 2000) it became more feasible to monitor microtubule dynamics in cultured mammalian neurons (Stepanova et al., 2003). Initially these experiments were conducted on primary neurons transiently transfected or infected with constructs or viruses encoding fluorescent protein-tagged end-binding proteins. More recently, the generation of mice carrying a transgene encoding YFP-tagged EB3 under the control of a promoter exclusively and consistently active in neurons opened the possibility to study neuronal microtubule dynamics during development *in vivo* (Kleele et al., 2014). In addition, primary neurons derived from these mice can be analyzed *ex vivo* under controlled conditions. In combination with a recently developed microtubule plus-end tracking software (Matov et al., 2010; Applegate et al., 2011), large scale quantitative microscopy investigations of a potential relationship between microtubule dynamics and growth cone behavior are now possible.

Assessment of dynamic changes of microtubule polymerization during mammalian growth cone migration faces several challenges: first, growth cone motility concurrently involves distinct spatial scales (molecular/macromolecular, subcellular domains, the entire growth cone). Secondly, microtubule polymerization rates are typically an order of magnitude higher than the speed of growth cone displacement (Geraldo and Gordon-Weeks, 2009). Furthermore, differential measurements and comparison of microtubule dynamic parameters in various spatial domains of individual growth cones (peripheral or central domains) might be important (Lowery and Van Vactor, 2009; Liu and Dwyer, 2014; Cammarata et al., 2016). Therefore, short-term morphological changes in migrating growth cones as well as microtubule polymerization dynamics may best be captured by the simultaneous acquisition of a number of different parameters over a range of spatiotemporal scales. This type of multiscale, multiparametric quantitative microscopy has recently been introduced, and employed to study the migration of non-neuronal cells (Lock and Stromblad, 2010; Lock et al., 2014; Kiss et al., 2015).

The aim of the present study was to detect potential correlations between microtubule polymerization and changes in growth cone migration. We performed a comprehensive, quantitative analysis of microtubule polymerization properties linked to morphological and migratory parameters of individual growth cones. This investigation was carried out using adult murine dorsal root ganglion (DRG) neurons which display robust axon growth in culture and are well established as a model of axon regeneration (Al-Ali et al., 2017). Based on the assumption that features of microtubule dynamics which are essential for proper growth cone migration might be altered in mutant neurons defective in growth cone guidance, we also included DRG neurons from mice lacking microtubule-associated protein (MAP) MAP1B.

MAP1B is one of the classical MAPs and is expressed at high levels in the developing brain (Gordon-Weeks and Fischer, 2000; Halpain and Dehmelt, 2006; Villarroel-Campos and Gonzalez-Billault, 2014). It is essential for proper brain development and axon guidance *in vivo* (Takei et al., 1997; Gonzalez-Billault et al., 2000; Meixner et al., 2000). *Ex vivo*, cultured primary neurons of various types display defects in axon growth, growth cone migration and growth cone response to attractive as well as repulsive axon guidance cues (Bouquet et al., 2004, 2007; Del Rio et al., 2004; Gonzalez-Billault et al., 2005; Stroissnigg et al., 2007; Meli et al., 2015). MAP1B binds to the lateral surface of microtubules and has been shown to stabilize them (Halpain and Dehmelt, 2006; Villarroel-Campos and Gonzalez-Billault, 2014). It can also interact with actin filaments (Halpain and Dehmelt, 2006; Villarroel-Campos and Gonzalez-Billault, 2014). Moreover, MAP1B directly influences microtubule dynamics, although the results reported in two different studies were partially contradictory (Tymanskyj et al., 2012; Tortosa et al., 2013). Of note, based on genetic manipulations diminishing but not completely abolishing MAP1B levels, MAP1B was reported to decrease (Tortosa et al., 2013) or increase (Tymanskyj et al., 2012) the rate of microtubule polymerization in axons of cultured primary neurons, while no differences in growth rate were found in growth cones. Thus, a further aim of the current study was to perform a detailed analysis of microtubule polymerization in growth cones of primary neurons completely lacking MAP1B (Meixner et al., 2000).

We found an association between growth cone size and basic parameters of microtubule polymerization. This correlation was also seen in MAP1B-deficient neurons which overall displayed reduced microtubule dynamics and an increase in growth cone size. Our findings indicate that microtubule dynamic parameters change as growth cones undergo the characteristic temporal size changes during migration. Moreover, our results suggest that effects of guidance cues, different substrata or genetic manipulations on growth cone microtubule dynamics must be interpreted in the context of the effects of such perturbations on growth cone size. On the other hand, no fundamental differences in microtubule dynamic parameters were found in extending vs. retracting growth cones. Our results indicate that the dynamicity of growth cone microtubules is a basic process which, in contrast to our expectations, is not strictly linked to the polarity of growth cone migration.

## Materials and methods

### Ethics statement

The current study does not contain *in vivo* experiments using live animals. Newborn mice were sacrificed by decapitation in compliance with the Austrian law regulating the use of animals in biomedical research, Tierversuchsgesetz, BGBl. Nr. 501/1989 and BGBl. I Nr. 162/ 2005. Since no experiments on live animals were performed, approval of the experiments by the Institutional Animal Care and Use Committee was not required according to the above cited law. Animals were housed at the in-house animal facility of the Max F. Perutz Laboratories which has been certified by the Austrian Federal Ministry of Science, Research and Economy (permit number BMWFW-66.006/0012-WF/II/3b/2014). Individual breeding pairs for the production of mice were held in breeding cages according to the guidelines of the Federation of European Laboratory Animal Science Associations (FELASA).

### Animals and DRG neuron culture

To obtain MAP1B-deficient and corresponding wild-type control mice carrying the YFP-EB3 transgene we crossed mice lacking the MAP1B gene (Meixner et al., 2000) with mice transgenic for the YFP-EB3 transgene (Kleele et al., 2014). For this we selected mouse line J045, because this line expresses the lowest levels of the transgene (Kleele et al., 2014). Both, the MAP1B^−/−^ and the YFP-EB3 transgenic lines were on the inbred C57BL/6 background. This breeding eventually yielded females heterozygous for the MAP1B deletion and homozygous for the YFP-EB3 transgene. These females were crossed with males heterozygous for the MAP1B deletion (but lacking the YFP-EB3 transgene) on the inbred 129P2 background. This breeding yields F1 hybrid offspring with identical genetic background, all carrying a single copy of the YFP-EB3 transgene and either wild-type, heterozygous or homozygous for the MAP1B deletion. Genotyping was performed by PCR with appropriate sets of primers and the genotype of mice used for DRG neuron preparations was confirmed again at time of sacrifice.

Dissociated adult DRG neuronal cultures were prepared from MAP1B^+/+, TG/0^ and MAP1B^−/−, TG/0^ mice 4–5 months of age. DRG neurons were isolated as described (Tonge et al., 1997) and were grown at a density of ~100 cells/cm^2^ on glass-bottom 35 mm ibidi imaging dishes coated with poly-l-lysine (10 μg/ml Sigma) and mouse laminin (20 μg/ml, Sigma)) in DMEM/F-12 medium (Gibco) supplemented with N2, L-glutamine and 20% horse serum for 20–24 h prior to live cell imaging.

### Live cell imaging

**Figure 1:**
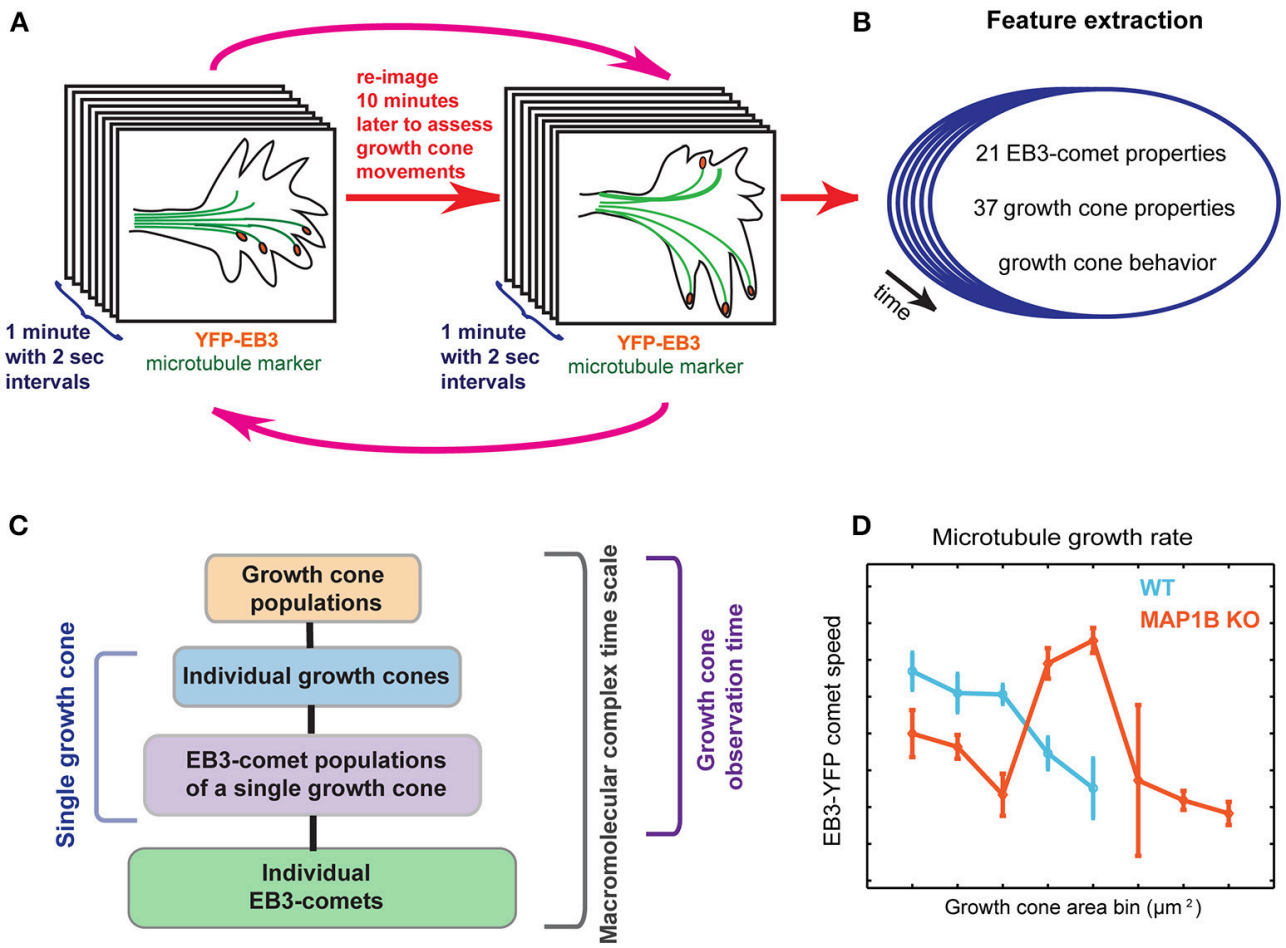
Graphical summary. **(A)** Imaging setup using multiple temporal scales. First, EB3-YFP expressing mouse DRG neuron growth cones are imaged for 1 min in 2-s intervals, providing the time resolution necessary to assess microtubule polymerization parameters. The same imaging procedure is repeated every 10 min over several cycles (magenta arrows), allowing visual scoring of net growth cone displacement (extension vs. retraction) of wild-type and MAP1B^−/−^ growth cones. **(B)** Multiple quantitative parameters of EB3-comets and growth cones are extracted, describing EB3-comet morphological, spatial, and dynamic properties (e.g., speed, lifetime) and growth cone morphology over time. Importantly, each of these properties is assigned to the corresponding individual growth cone and the observed behavior of the particular growth cone (extension vs. retraction). **(C)** Spatial and temporal hierarchy of the dataset. Parameters are simultaneously recorded across all of the indicated scales. Statistical measurements of entire growth cone populations are the top level of this data hierarchy, followed by the level of individual growth cones. Each of the growth cones contains a group of YFP-EB3 comets, the properties of which are changing over time. Thus, the next data level captures aggregate statistics of the growth cone comet population, for all time points (termed EB3-comet time scale, i.e., 2-s intervals), for each growth cone. The properties of individual EB3-comets are also recorded, thereby providing the maximal spatial data disaggregation. Images are acquired for 30 min (growth cone observation time). Comet data may also be disaggregated to reflect instantaneous dynamics at the 2-s sampling frequency, across all spatial scales. **(D)** Data stratification according to growth cone size reveals associations between microtubule dynamicity and growth cone area.

High-resolution, multiscale imaging was performed on an inverse microscope equipped with a Yokogawa CSUX1A1 Nipkow spinning disc unit using a PlanApochromat 63 × /1.4 NA oil-immersion objective. Images were acquired for 30 min repeating the 33 × 2 sinterval cycles (as described in Figure 1) with a pixel resolution of 0.211 μm. The culture medium was replaced with phenol-red free DMEM/F12 medium (Gibco) 1 h prior to imaging, and environmental conditions of 37°C and 5% CO_2_ were maintained throughout the process.

### Image analysis

YFP-EB3 comets were automatically segmented using the plusTipTracker software (Matov et al., 2010; Applegate et al., 2011) with the following tracking settings: Search Radius Range: 1.5-6 pixels; Minimum Track Length: 3 frames; Maximum Gap Length: 3 frames; Maximum Shrinkage Factor: 1.4; Maximum Forward Angle: 35°; Maximum Backward Angle: 15°; Fluctuation Radius: 1.25. The analyses included only EB3-comets tracked for a minimum of 3 frames (6 s) and disappearing for a maximum of 3 frames (6 s), to minimize false positives. Most of these tracking parameters were defined based on a study in *Xenopus laevis* growth cones (Stout et al., 2014). Comets were labeled and tracked individually (Figure S1) throughout their lifetime (from first until last detection). Only comets appearing and disappearing within the imaging interval (about 1 min) were included in the comet distance to growth cone front at comet birth or comet death analysis. Comet intensity was not analyzed as part of this study.

### Growth cone segmentation

Individual growth cones were selected using the plusTipTracker ROI (region of interest)-selection tool. Growth cone outlines were segmented based on cytoplasmic YFB-EB3 background signal using a custom-written MATLAB tool. Briefly, growth boundaries were identified using a combination of Sobel edge detection method and other morphological pixel operations (dilation, filling holes, erosion) of the MATLAB Image Processing Toolbox. Properties describing growth cone morphology were extracted using the “regionprops” MATLAB function. In order to confirm that cytoplasmic YFP-EB3 background-based segmentation is a good approximation of the total growth cone area, a number of growth cones were labeled with SiR-actin in live neurons, imaged and segmented in parallel based on YFP-EB3 and SiR-actin using the same methods. The segmentation results of these different channels are consistent (Figure S2).

Growth cone midline was determined based on distance transform of the growth cone segmentation mask, using the assumption that points on the midline have maximal distance from the lateral growth cone boundaries. Then, growth cone front was automatically calculated as boundary coordinates corresponding to a 1:1.7 division rule of the midline. Comet localization with respect to these growth cone landmarks was defined as the minimal Euclidean distance of the comet coordinates from the segmented curves (John D'Errico; https://de.mathworks.com/matlabcentral/fileexchange/34869-distance2curve).

### Dataset summary and statistical analysis

The final dataset contains measurements obtained in 6 wild-type and 4 MAP1B-null biological replicates, respectively. Properties were extracted on different spatial (individual growth cones and their YFP-EB3 comets) and temporal (minutes vs. seconds time resolution) scales, integrated on a single growth cone level. Microtubule polymerization parameter statistics (e.g., growth rate, localization with respect to growth cone) were assembled based on the trajectories of individual EB3-comets, while growth cone statistics (e.g., number and density of comets, median speed of microtubule polymerization) were calculated per growth cone. Altogether, the dataset contains 26060 time-resolved observations of 783 growth cones and 141096 time-resolved observations of 18169 individual EB3-comets. In total, 37 variables are associated with the growth cone-level observations, and 21 variables with EB3-comet level observations.

95% confidence intervals for the medians were determined based on MATLAB's “boxplot” function: lower CI: $q2-1.57^{*}\frac{IQR}{\sqrt{n}}$; upper CI: $q2+1.57^{*}\frac{IQR}{\sqrt{n}}$ where q2 is the median (50th percentile), IQR is the interquartile range (difference of 75 and 25 percentiles) and n corresponds to the number of observations. Wilcoxon- rank sum tests were used for pairwise comparisons of wild-type and MAP1B^−/−^ growth cone and comet properties. In the comparisons of extending vs. spontaneously retracting growth cones—due to the low number of retracting growth cones per growth cone area bin - *p*-values have been determined via bootstrap sampling. All *p*-values were adjusted for multiple comparisons with the Holm-Bonferroni correction method.

Unsupervised multivariate clustering was performed using the principal component analysis tool of the Cell Adhesion and Migration Analysis Toolbox (Shafqat-Abbasi et al., 2016).

### Growth cone area continuous sampling and stratification

Time-resolved growth cone observations were sorted according to increasing growth cone area. Using a moving window sampling (window size: 300, overlap: 220 observations), bootstrapped (resampling with replacement) median property values per sampling window were calculated as described (Kiss et al., 2015). The bootstrapped results were re-aggregated according to equidistant growth cone area bins (20 μm^2^ incremental steps). The consecutive usage of fine- and coarse-grained sampling allowed to determine that the observed relationships between microtubule polymerization parameters and growth cone size are not due to binning artifacts.

### Differential probability calculations

Differences of YFP-EB3 comet properties in adjacent growth cone area bins were calculated as described (Kiss et al., 2015). Briefly, histograms having the same bin number and bin width between the minima and 97th percentile of YFP-EB3 comet features for each growth cone area bin were calculated. Probability distributions were obtained by dividing the histogram values of each feature by the total number of comet observations within the particular growth cone area bin. Proportional differences were calculated by subtracting MAP1B^−/−^ proportional distributions from wild-type proportional distributions of the corresponding wild-type growth cone area bins.

## Results

DRG neurons were obtained from adult mice carrying a transgene driving the neuron-specific expression of YFP-EB3 (Kleele et al., 2014). This system avoids potential problems of transient transfection in that YFP-EB3 is expressed in all neurons at low levels. YFP-EB3 serves as fluorescent marker of actively polymerizing microtubule plus ends (Akhmanova and Steinmetz, 2008). Growing plus ends are visible as “comets” in live growth cones. For the purpose of this report, the number, speed, lifetime, and localization of comets will be used synonymously for the number of actively growing microtubules and the rate, duration and localization of microtubule polymerization, respectively.

We simultaneously extracted parameters describing growth cone behavior (extension vs. retraction) and organization (e.g., growth cone morphology, comet parameters). YFP-EB3 comets were automatically segmented and tracked using the plusTipTracker (Matov et al., 2010; Applegate et al., 2011), growth cones were segmented using custom-written tools (see Materials and Methods, Figure S1, S2 and Movies S1–2). Growth cone behavior (extension vs. retraction) was determined based on growth cone tip displacement between 10-min intervals. Altogether, quantitative properties of 783 migrating (wild-type and MAP1B^−/−^) growth cones and microtubule polymerization properties of their associated 18169 YFP-EB3 comets were extracted using imaging at two different time scales (Figure 1, Figure S3 and Tables S1, S2).

**Figure 2:**
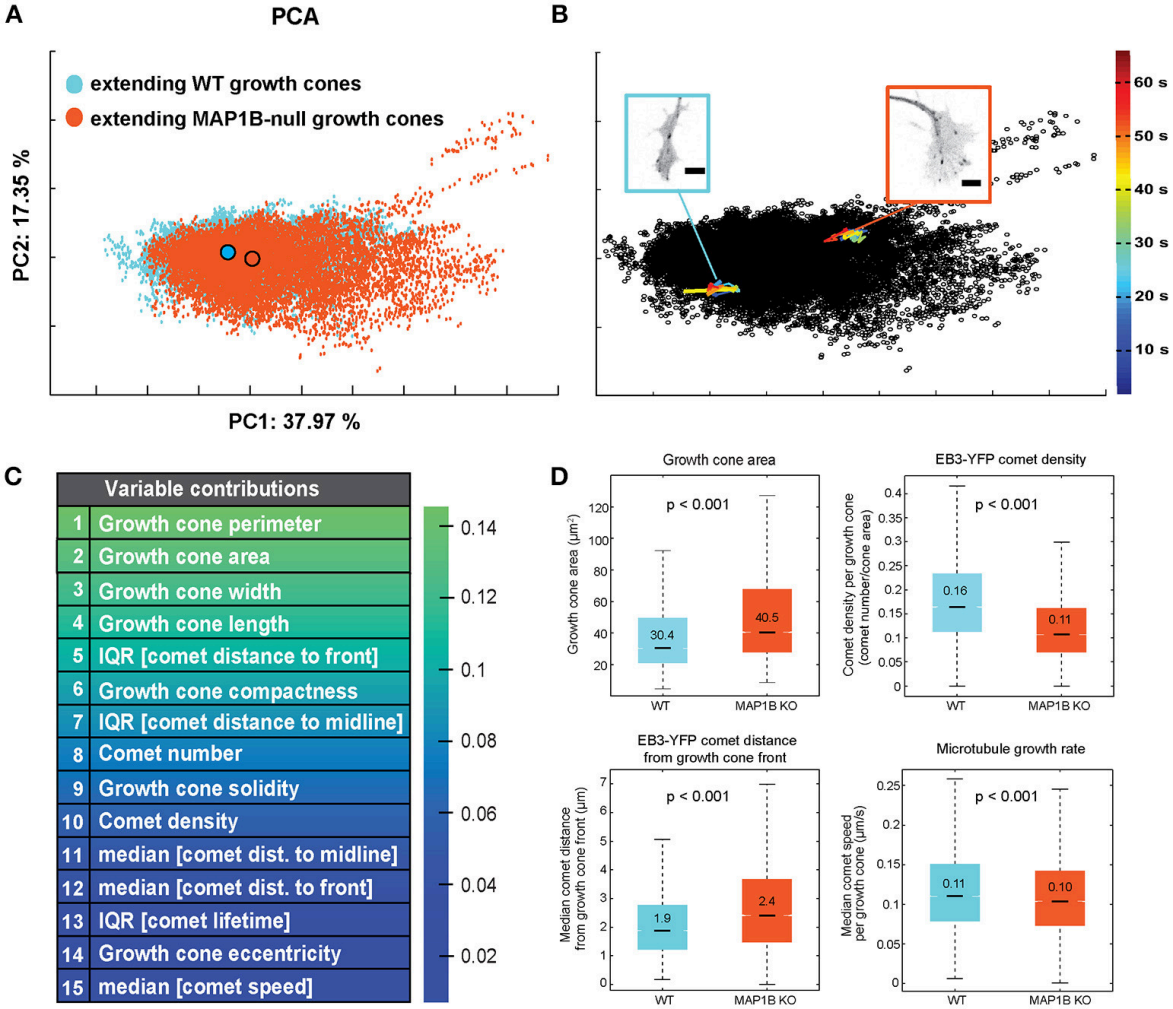
Wild-type and MAP1B-null extending growth cones mainly vary in growth cone morphology. **(A)** PCA-based clustering of wild-type (blue) and MAP1B-null (orange) extending growth cones. Principal component 1 (PC1) and PC2 are displayed, percentages show proportion of total variance included per PC. Outlined circles indicate the population centers of mass. Note the substantial overlap between the two growth cone subpopulations in the multivariate feature space. Number of growth cone observations: wild-type: 13553, MAP1B-null: 11443. **(B)** Each dot of the PCA scatterplot corresponds to a single time point (2 s) observation of an individual growth cone. Multivariate trajectories over time (33 time points each) of a wild-type (blue) and a MAP1B-null (orange) growth cone are overlaid on the PCA plot shown in **(A)**. Image insets show single frames of the corresponding growth cone image sequences. Color scale corresponds to the development of the trajectories over time in the PCA-space. Scale bars: 5 μm. **(C)** List of top 15 growth cone and EB3-comet properties ranked by their contribution to PC1 (PCA coefficient values). **(D)** Comparison of growth cone area, EB3-YFP comet density, EB3-YFP comet distance to growth cone front and microtubule growth rate between wild-type and MAP1B-null extending growth cones. In the box plots, black lines and numbers indicate the median values; boxes show the interquartile range (IQR, Q3-Q1), notches on the boxes indicate 95% confidence intervals for the median, whiskers mark the values of Q1(25th percentile) −1.5*IQR and Q3(75th percentile) + 1.5*IQR, respectively. The indicated *p*-values were obtained from Wilcoxon rank-sum tests. Number of observations: as in **(A)**.

First, we assessed the multivariate combination of microtubule polymerization parameters and growth cone morphological features of spontaneously extending wild-type and MAP1B^−/−^ growth cones using principal component analysis (PCA). PCA-based clustering of extending growth cones revealed a substantial overlap between wild-type and MAP1B^−/−^ growth cone populations in the PCA space (Figure 2A). This overlap is most likely due to considerable variability between individual growth cones in each group, as multivariate trajectories of individual growth cones are confined to a relatively small area in feature space (Figure 2B). PCA analysis identified growth cone size-related and morphological features as the main contributors of the total variance found in extending growth cones (Figure 2C). These parameters are significantly different in univariate comparisons of wild-type and MAP1B-null extending growth cones (Figure 2D). Notably, growth cones can reach a much larger size in MAP1B^−/−^ neurons. Nevertheless, the majority of MAP1B-null growth cones (90%) correspond in size to their wild-type counterparts. This overlap is also discernible from cumulative distribution functions (CDF) of growth cone area for wild-type and MAP1B^−/−^ growth cones (Figure S4A).

As one might expect as a result of deleting a MAP, parameters associated with microtubule polymerization display significant differences between wild-type and MAP1B-null growth cones including number, density and the spatial distribution of comets. Aggregate comparisons (including all extending growth cones) of these properties between wild-type vs. MAP1B^−/−^ DRG neurons revealed a 33% increase in growth cone size consistent with previous reports (Gonzalez-Billault et al., 2002; Tortosa et al., 2013), a 31% decreased density of comets and a 26% increased distance of comets from the growth cone front (Figure 2D). In contrast, the difference in microtubule growth rate, when averaged over all growth cones of each group, appeared to be modest. In wild-type growth cones we measured a median comet speed of 0.11 μm s^−1^ which is in agreement with previous measurements in DRG neurons (Harkcom et al., 2014; Kleele et al., 2014). In MAP1B^−/−^ growth cones the aggregate median comet speed was slightly lower at 0.10 μm s^−1^, a 9% decrease (Figure 2D).

The finding that growth cone size-related features (perimeter and area) are substantially different in wild-type and MAP1B-null growth cones (Figure 2D) suggested that parameters describing growth cone morphology and microtubule polymerization should be re-examined in the context of growth cone size. The unprecedented amount of quantitative data collected from several hundreds of growth cones and their tens of thousands of comets provided the unique possibility to explore these relationships in detail. We stratified the dataset according to growth cone area, choosing 8 bins of 20 μm^2^ ranging from 10 to 170 μm^2^. The first 5 of these bins (size classes of 10-110 μm^2^) contain observations of wild-type as well as MAP1B-null growth cones, whereas larger growth cones (110-170 μm^2^) could only be observed in MAP1B-null neurons (Figures S4B,C). Thus, for the subsequent analyses a direct comparison between wild-type and MAP1B-null growth cones could only be completed in the first 5 size classes. However, in the next data presentations we will also include the measurements obtained in very large MAP1B-null growth cones to explore whether parameter values continue to display size-dependence in very large growth cones albeit only for MAP1B-null neurons.

We first examined size-dependent morphological differences between wild-type and MAP1B-null growth cones, because growth cone length and width featured prominently in the PCA (Figure 2C). As expected, growth cone length and width increased in monotonic fashion with growth cone size, both in wild-type and MAP1B-null growth cones (Figures S5A,B). However, MAP1B-null growth cones were significantly shorter and wider compared to their wild-type counterparts in almost all size classes. This is also reflected in the lower values of growth cone eccentricity (Figures S5C,D). For both wild-type and MAP1B-null neurons, growth cone eccentricity decreased with growth cone area. However, in wild-type growth cones this decrease is only present between the two smallest (10–30 and 30–50 μm^2^) size groups, indicating that as growth cones increase in size, their length and width increase proportionally. In contrast, MAP1B-null growth cones display a strong decrease in eccentricity with the gain in size resulting from a stronger increase in width relative to length.

**Figure 3:**
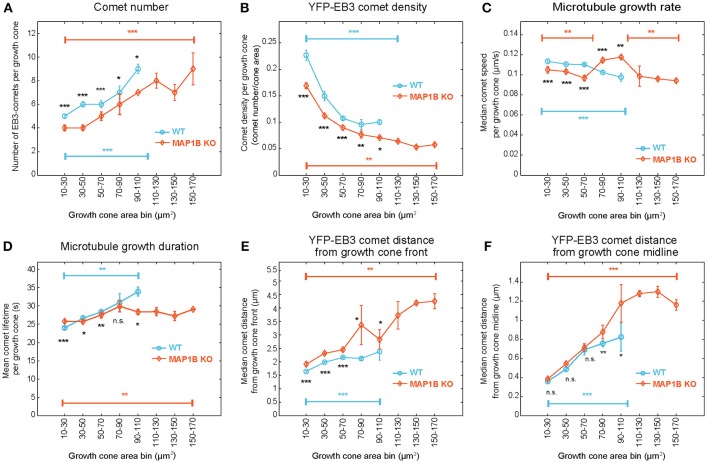
EB3-comet associated features scale with growth cone size both in wild-type and MAP1B-null growth cones. Wild-type (blue) and MAP1B-null (orange) growth cone populations were stratified according to increasing growth cone area bins (interval: 20 μm^2^). The largest size bins (110–170 μm^2^) only contain MAP1B-null growth cones. EB3-YFP comet number increases **(A)** while comet density **(B)** and microtubule growth rate **(C)** are decreasing with increasing growth cone size, and significantly differ between each wild-type and MAP1B-null growth cone area bins. Microtubule growth duration **(D)**, comet distance from growth cone front **(E)** and comet distance from growth cone midline **(F)** are increasing with increasing growth cone size. Median property values and their 95% confidence intervals are displayed. Number of observations is displayed in Figure S4. *p < 0.05, **p < 0.01, ***p < 0.001, n.s., non-significant; according to Wilcoxon rank-sum test.

Next we aimed to determine whether growth cone size has an impact on microtubule polymerization parameters. Stratification of the dataset according to growth cone area not only revealed that many microtubule polymerization parameters differ between wild-type and knockout growth cones depending on growth cone area (Figure 3), but also that these parameters change with increasing growth cone size. In wild-type growth cones, the number of comets (Figure 3A), the duration of microtubule growth (Figure 3D) and the comet distance from the growth cone front (Figure 3E) and from the growth cone midline (Figure 3F) all increase with increasing growth cone size. In contrast, comet density (Figure 3B) and the rate of polymerization (Figure 3C) decrease with increasing growth cone size. In MAP1B-null growth cones, as expected from the median values shown in Figure 2D, comet density is lower and comet distance to growth cone front is higher than in wild-type growth cones (Figure 3). In general, the size-dependence observed in wild-type growth cones is also seen in MAP1B-null growth cones. One exception to this is comet lifetime which is longer in small and shorter in large MAP1B-null growth cones, reaching a plateau in growth cones of 70–90 μm^2^ (Figure 3D). Moreover, comet speed is ~ 10% lower in small (area 10–70 μm^2^) and ~ 15% higher in larger (area 70–110 μm^2^) MAP1B-null growth cones compared to wild-type growth cones of the corresponding size (Figure 3C). These differences between wild-type and MAP1B-null growth cones and the non-monotonic size dependence of microtubule growth rate could not have been inferred from the aggregate data shown in Figure 2D, which only revealed a 9% decrease in comet speed in MAP1B-null growth cones. This 9% decrease on aggregate may be due to the fact that most growth cones of wild-type and MAP1B-null neurons are in the 10-70 μm^2^ size range (Figures S4A,B). In small (area 10–70 μm^2^) and large (area above 110 μm^2^) MAP1B-null growth cones the steady decrease in comet speed with growth cone size mirrors what we found in wild-type growth cones (area 10–110 μm^2^). Continuous sliding window sampling and randomized binning of growth cone area confirmed that the above detected associations between growth cone size and microtubule dynamicity are not artifacts introduced by our choice of growth cone area bins (Figure S6).

**Figure 4:**
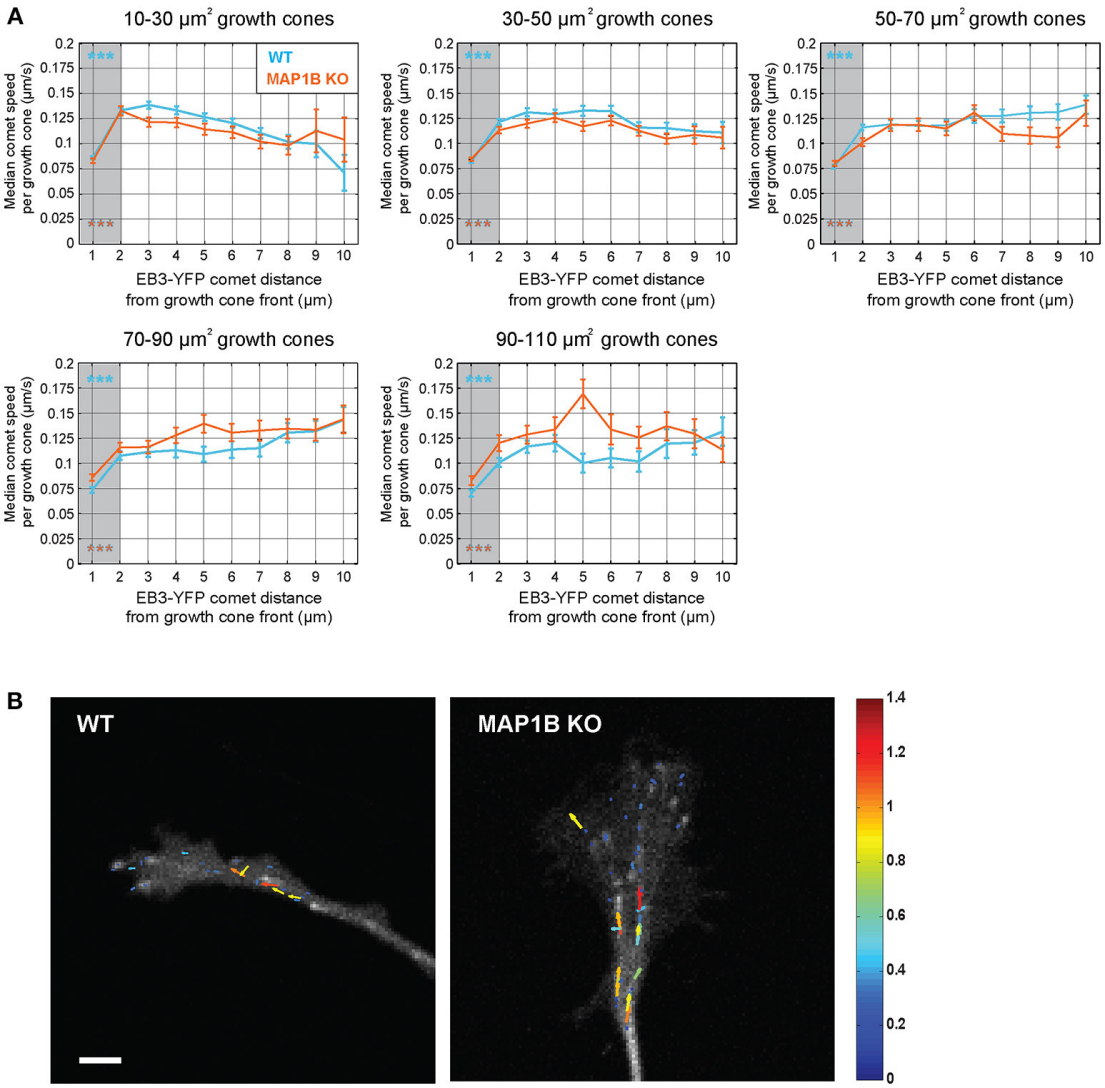
Comet speed decreases near the growth cone front.**(A)** Instantaneous speed of individual YFP-EB3 comets was grouped according to distance to growth cone front in wild-type and MAP1B-null growth cones. Error bars represent the median speed value and 95% confidence interval of comets located within a specific distance interval (e.g., below 1 μm, between 1 and 2 μm, etc.) from the growth cone front. Comets being closer than 2 μm to the growth cone front are slower than comets being further away. ***p < 0.001, Wilcoxon rank-sum test. **(B)** Quiver plots showing representative examples of comet movements in wild-type and MAP1B-null growth cones. Color and length of the individual arrows reflects instantaneous comet speed (displacement over 2 s). Scale bar, 5 μm.

Previous reports demonstrated that the rate of microtubule polymerization is reduced near the cell edge of epithelial and endothelial cells (Kumar et al., 2009; Matov et al., 2010; Applegate et al., 2011; Nishimura et al., 2012). Here we wanted to test whether a similar decrease of comet speed can be observed near the growth cone front and whether this is influenced by growth cone size. Stratification of instantaneous comet speed data according to comet distance to growth cone front revealed a reduction in comet speed near the front and that this was independent of growth cone size and the presence of MAP1B (Figure 4). However, in larger growth cones (70–110 μm^2^) comet speed was higher near the leading edge (and elsewhere) in MAP1B-null growth cones compared to wild-type, reflecting the higher values for median comet speed found in large MAP1B-null growth cones (Figure 3).

**Figure 5:**
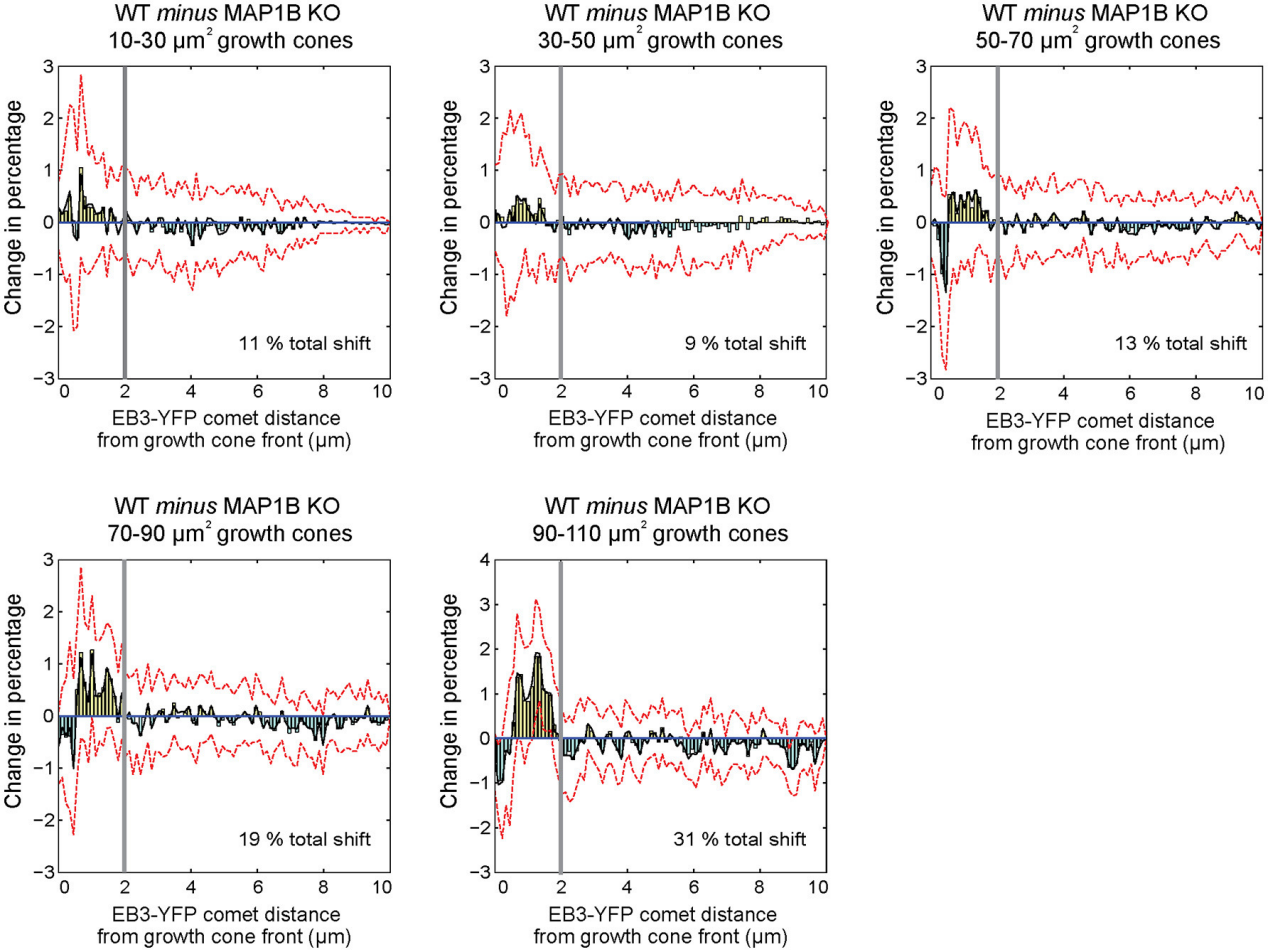
Differential probability distributions reveal growth cone area-independent, but selective influence of MAP1B over comet distance to growth cone front. Differential probability plots indicate detailed differences between distributions. Briefly, histograms with equal number, and size of bins were created from comet property values for each growth cone area group. Histogram values were divided by the total number of observations within each area bin, to obtain probability distributions. In order to monitor the effect of absence of MAP1B, probability distributions were subtracted from each other (*WT minus MAP1B-null*). Bars above 0 indicate a proportional increase within the specified intervals of wild-type growth cones, while bars below 0 show a proportional decrease (in wild-type growth cones as compared to MAP1B-null growth cones of the same area bins. Black lines indicate the bootstrapped median of differential probabilities, and red dashed lines indicate the 5 and 95 percentiles of the bootstrapped distributions. Note that distributions of EB3-comet distance to growth cone front display structured changes in with a switch point located ~ 2 μm from the growth cone front (marked by thick gray vertical lines), and that this specific switch point is found in each growth cone area bin. The analysis was based on at least 4,800 individual EB3-comet observations per growth cone area bin.

One of the differences between wild-type and MAP1B-null growth cones is the increased comet distance to growth cone front (Figure 3E). To clarify the underlying reason and structure of this increase, we generated differential probability distributions by subtracting the normalized number of EB3-comet observations of MAP1B-null from wild-type comet observations, again stratified according to growth cone area (Kiss et al., 2015). This shows the differences between wild-type and the corresponding MAP1B-null growth cone size classes grouped by comet distance from growth cone front. We found that proportionally fewer comets are found in MAP1B-null growth cones in a zone spanning the first 2 μm from the growth cone front (Figure 5). This difference to wild-type growth cones is independent of growth cone area, as it is present across all area bins. Thus, the width of the zone containing proportionally less comets in MAP1B-deficient growth cones is similar in all growth cone size classes analyzed. However, in larger (50–110 μm^2^) but not smaller MAP1B-deficient growth cones we observed a slight increase in the proportion of comets very close to the growth cone front (within 1 μm).

Visual inspection of the time-lapse analysis of comet movement indicated, that most microtubule polymerization proceeded in anterograde direction with only few cases of comets moving sideways or in retrograde direction (Movie S3). This was true for both wild-type and MAP1B-null growth cones. Quantitative analysis of the angular distribution of comet paths confirmed this supposition. For this analysis we determined the paths between beginning and end positions of individual comets and analyzed their angles relative to the growth cone midline in growth cones of different size classes. The majority of comets (between 82 and 88%) moved in anterograde direction toward the leading edge with small percentages of comets moving retrogradely or at an angle with respect to the growth cone midline (Figure S7). These percentages were similar in growth cones of all size classes. In MAP1B-null growth cones the percentage of anterograde comets was slightly reduced to between 74% in small and 85% in large growth cones with a concomitant increase in retrograde comets.

**Figure 6:**
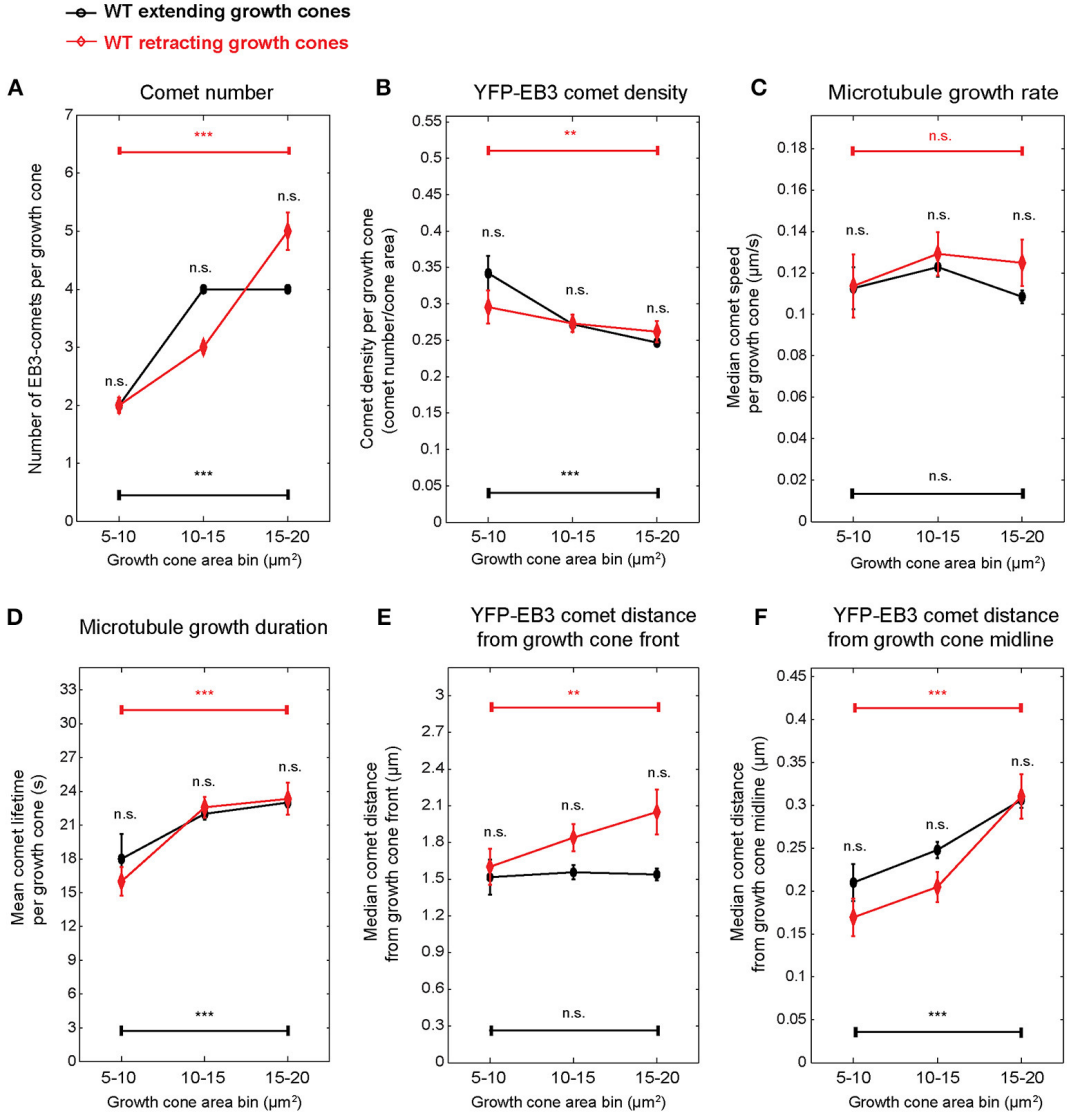
Microtubule polymerization features show no difference between wild-type extending and retracting growth cones. Spontaneously extending and retracting wild-type growth cones were stratified according to growth cone area, and the microtubule growth parameters presented in Figure 3 were compared between them. Only growth cones smaller than 20 μm^2^ were included. Median values and 95% confidence intervals are shown for comet number **(A)**, comet density **(B)**, microtubule growth rate **(C)**, microtubule growth duration **(D)**, comet distance from growth cone front **(E)**, and comet distance from growth cone midline **(F)**. No significant differences were found between extending and retracting growth cones of the same growth cone size category. **p < 0.01, ***p < 0.001, n.s., non-significant; according to Wilcoxon rank-sum test.

The experimental setup used here, consisting of measurements of growth cone and comet parameters in individual growth cones for 1 min in 10-min intervals over 30 min allowed us to associate growth cone and comet parameters with individual growth cone behavior (extension vs. retraction). Typically, extension or retraction lead to a displacement of the growth cone centroid by 2.5 μm in 10 min. PCA-based unsupervised clustering of extending and retracting wild-type growth cones exposed again growth cone size-related parameters (Figure S8). Therefore, we stratified the data again according to growth cone area. Since 80% of retracting growth cones are found in a size range of 5–20 μm^2^ (Figure S8), we applied a more refined binning focusing on small growth cones. Using this refined size stratification, we found that the percentage of retracting growth cones increases from 14% in the 15 to 20 μm^2^ size class to 20% in the 10 to 15 μm^2^ and 50% in the 5 to 10 μm^2^ size classes (Figure S8) indicating an inverse correlation between growth cone size and the propensity to retract. In small extending growth cones (Figure 6)—as expected from the data presented in Figure 3—comet number, density, distance to front, distance to middle, and lifetime are size-dependent. Retracting growth cones displayed similar relationships with the individual parameters not significantly different from extending ones. There was no significant difference in comet speed in extending or retracting growth cones ranging from 5 to 20 μm^2^.

## Discussion

Cytoskeleton rearrangements and in particular the regulation of microtubule dynamics have long been recognized to play key roles in neuronal growth cone migration and steering (Lowery and Van Vactor, 2009). To obtain an accurate picture of local and temporal regulation of growth cone microtubule dynamics, direct quantitative measurement of dynamic parameters in live growth cones is necessary. This has been greatly facilitated by fluorescent labeling of microtubule plus end-binding proteins EB1 and EB3 (Mimori-Kiyosue et al., 2000; Stepanova et al., 2003). The development of automated microtubule plus-end tracking software (Matov et al., 2010; Applegate et al., 2011) and the generation of transgenic mice expressing a YFP-EB3 fusion protein in all neurons (Kleele et al., 2014) were additional technical advances facilitating rapid quantitative analysis of mammalian growth cone microtubule dynamics. For the current study we employed a novel combination of these advances. This enabled us to obtain an unprecedented large dataset comprising spatial, temporal and kinetic information of microtubule polymerization linked to morphological and migratory characteristics of hundreds of individual growth cones. The considerable size of the dataset permitted stratification and re-sampling according to different variables to reveal patterns and correlations which had not been seen previously. To our knowledge, the current study represents the first comprehensive quantitative analysis of microtubule polymerization in growth cones of primary mammalian neurons.

### Microtubule polymerization parameters change with size of the growth cone

One of the key findings of our study is that a number of microtubule polymerization parameters display changes associated with the increasing growth cone size. Migrating growth cones are highly dynamic structures that undergo changes in size at a time scale of tens of minutes to hours (Dent and Gertler, 2003; Lowery and Van Vactor, 2009; Goodhill et al., 2015). Microtubule dynamics are typically sampled at a much shorter time scale (500 ms to 2 s) and hence will represent a snapshot of microtubule dynamics at a given growth cone size.

To evaluate a potential correlation between growth cone size and microtubule polymerization parameters we selected wild-type growth cones that extended over 20 min and grouped them in 5 size classes ranging from 10 to 110 μm^2^ (Figure 3). Of note, the speed of migration of these growth cones was independent of growth cone size at about 0.25 μm per min. We found that as the growth cone size increases, the number of comets increases as well, albeit not to the extent that it could compensate for the increase in growth cone area. Thus, comet density decreases with size. Likewise, the rate of microtubule polymerization decreases in larger growth cones while the duration of polymerization increases. While our analysis cannot reveal the underlying molecular mechanism or a potential causal relationship between microtubule polymerization parameters and growth cone size, it clearly demonstrates the necessity of the interpretation of microtubule dynamics parameter values in the context of individual growth cones. This may be of particular importance in the most frequent experimental scenarios where effects of environmental conditions or other perturbations on growth cone microtubule dynamics are to be determined and compared. Various substrata on which neurons are plated (Burden-Gulley et al., 1995), treatments with axon guidance cues (Lowery and Van Vactor, 2009) or genetic manipulations (this study, see below; Paglini et al., 1998; Gonzalez-Billault et al., 2002; Tortosa et al., 2013) all can alter growth cone size and thereby might indirectly influence microtubule dynamics, in addition to their potentially more direct effects, for example through binding to microtubules or modulating signal transduction pathways which also regulate microtubule dynamics. The large scale multiparametric analysis performed here can help to separate the direct from the indirect, size-related effects of such perturbations on microtubule dynamics.

### Local differences of microtubule polymerization parameters in growth cones

Our analysis also revealed several features of growth cone microtubule polymerization that are independent of growth cone size. Microtubule polymerization is observed throughout the growth cone with about 85% of comets proceed anterogradely toward the leading edge. The rate of microtubule polymerization is higher in proximal parts of the growth cone and declines within 1–2 μm of the leading edge whereas the duration of polymerization is similar in the 2-μm zone and in more proximal parts of the growth cone (not shown). Our finding that the microtubule polymerization rate is reduced close to the leading edge is reminiscent of what has been reported previously for non-neuronal cells (Kumar et al., 2009; Matov et al., 2010; Applegate et al., 2011; Nishimura et al., 2012) and highlights the 2-μm zone as a special compartment in the growth cone. A potential reason for the apparent reduction in microtubule polymerization rate could be actin retrograde flow, which is active at the periphery and could enforce the retrograde movement of growing microtubules, diminishing the apparent polymerization rate (Schaefer et al., 2008; Lowery et al., 2013; Turney et al., 2016). In non-neuronal cells it was also shown that polymerizing microtubule plus ends, after reaching the cell periphery, undergo frequent transitions between short phases of growth and shrinkage (Komarova et al., 2002; Straube and Merdes, 2007). It was proposed that this dynamic behavior serves to accommodate the rapidly changing shape of the leading edge of migrating cells (Komarova et al., 2002). As we show here, this might also apply to extending growth cones. Although we could not directly visualize microtubule shrinkage with our experimental setup, the observation of many short comet tracks very close to the growth cone leading edge is consistent with the above mentioned frequent transitions between short phases of microtubule growth and shrinkage at the periphery.

### Microtubule polymerization dynamics are similar in extending and retracting growth cones

Given the importance of a dynamic microtubule network for growth cone migration and steering (Lowery and Van Vactor, 2009; Dent et al., 2011) we sought to determine whether spatial and/or kinetic microtubule polymerization parameters are fundamentally different in spontaneously extending vs. retracting growth cones. We found that basic parameters of microtubule polymerization such as rate and duration of growth or number and density of growing microtubules are not significantly different in retracting growth cones. Thus, we propose that the dynamicity of microtubules which has been shown to be essential for growth cone migration and steering (Lowery and Van Vactor, 2009; Dent et al., 2011) constitutes a basic process that enables the necessary microtubule rearrangements during extension and retraction. Growing microtubule plus ends can be viewed as mobile assembly platforms for protein complexes termed +TIP networks (Akhmanova and Steinmetz, 2008). Thus, while microtubule polymerization as a basic process could ensure that these mobile assembly platforms sample most of the growth cone compartments, other mechanisms, for example locally restricted phosphorylation of individual +TIP network components (Akhmanova and Steinmetz, 2008) in response to attractive or repulsive axon guidance cues, could determine the protein composition and the function of the local +TIP network and thereby promote extension or retraction. While our study indicates that regulation of microtubule polymerization is not necessary to determine whether a growth cone extends or retracts, we cannot exclude that stimuli such as axon guidance cues promoting extension or inducing retraction also involve changes in microtubule dynamicity.

### Lack of MAP1B leads to changes in microtubule polymerization dynamics in a growth cone size-dependent manner

The effect of downregulation of MAP1B on microtubule dynamics in primary neurons has been addressed in two previous studies (Tymanskyj et al., 2012; Tortosa et al., 2013). For these experiments the authors used embryonic hippocampal (Tortosa et al., 2013) or cortical (Tymanskyj et al., 2012) neurons in which MAP1B levels were downregulated, but not completely abolished (Gonzalez-Billault et al., 2000; Tymanskyj et al., 2012; Tortosa et al., 2013). The two studies agree in that they find a slight increase in retrograde comets in axons (Tymanskyj et al., 2012) and growth cones (Tortosa et al., 2013) which we also find here (Figure S7). However, while Tymanskyj et al. report a decrease in the rate of microtubule polymerization after downregulation of MAP1B expression, Tortosa et al. find an increase in comet speed in axons, but not in growth cones. Here we extensively analyzed the rate of microtubule polymerization in growth cones of adult DRG neurons completely lacking MAP1B (Meixner et al., 2000). Comparing microtubule polymerization measurements averaged over all growth cones (irrespective of their size) we found a modest decrease (9%) in comet speed in MAP1B-deficient growth cones (Figure 2) which could be missed in a less extensive analysis. However, stratification of the data according to growth cone area provides a very different insight and reveals that comet speed scales non-monotonously with area. In smaller growth cones (10–70 μm^2^) representing the majority of growth cones, comet speed is reduced in a MAP1B-null background. In contrast, comet speed is significantly increased in MAP1B-null growth cones of 70–110 μm^2^, demonstrating the importance of taking growth cone size into account. The reason for the non-monotonous scaling of comet speed with growth cone size is not clear at this point, but it is reminiscent of observations made in a human osteosarcoma cell line (Nishimura et al., 2012). In these cells downregulation of MAP1B causes a slight increase in the percentage of slow comets, similar to the slight reduction in average comet speed we found in smaller growth cones. In contrast, when osteosarcoma cells express a constitutively activated Rac1 protein, the downregulation of MAP1B causes a strong increase in the percentage of fast comets, analogous to our observations in large growth cones. It is tempting to speculate that larger growth cones differ significantly from smaller ones in the regulation of their cytoskeleton and that this could switch the effect the deletion of MAP1B will have on microtubule polymerization.

MAP1B could modulate microtubule dynamics in a number of ways, for example by directly affecting microtubule stability (Schoenfeld and Obar, 1994; Gordon-Weeks and Fischer, 2000) or polymerization (Pedrotti and Islam, 1995; Noiges et al., 2002). However, most of our observations in MAP1B-null growth cones are consistent with the model put forward by Tortosa et al. (2013) suggesting that MAP1B can sequester EB1 and EB3 and that in the absence of MAP1B the concentration of active EB1/3 in growth cones is increased (Tortosa et al., 2013). It has been shown that downregulation of EB1 in neuroblastoma cells decreases the rate and increases the duration of microtubule growth (Stepanova et al., 2010). In contrast, deletion of MAP1B will increase the availability of EB1/3 (Tortosa et al., 2013) and should have the opposite effects. Indeed, as is best seen in larger growth cones (70–110 μm^2^) in the absence of MAP1B the rate of polymerization is increased, the duration is decreased (Figure 3). Moreover, EB1 can inhibit initiation of microtubule polymerization from stabilized microtubule seeds *in vitro* (Wieczorek et al., 2015). Consistent with an increase of EB1/3 in the absence of MAP1B, the number of comets is reduced in MAP1B-null growth cones (Figure 3).

One consistent difference in MAP1B-null growth cones is the reduction of microtubule polymerization activity at the leading edge. Thus, the average comet distance to growth cone front is higher (Figure 3) and the proportion of comets in a zone spanning the first 2 μm from the leading edge is lower in MAP1B-deficient growth cones (Figure 5). This raises the question as to how MAP1B might promote microtubule polymerization close to the leading edge. MAP1B has been shown to bind both microtubules and actin filaments (Pedrotti and Islam, 1996; Tögel et al., 1998; Bouquet et al., 2004). In growth cones, MAP1B localizes in the cytoplasm as well as on microtubules and actin filaments (Bouquet et al., 2004; Stroissnigg et al., 2007). Co-localization with F-actin has been found predominantly at the leading edge (Bouquet et al., 2004). An intriguing possibility is that MAP1B facilitates microtubule polymerization close to the leading edge by acting as a link between F-actin and microtubules as has been proposed previously (Bouquet et al., 2004; Lowery and Van Vactor, 2009).

Extending previous findings (Gonzalez-Billault et al., 2002; Tortosa et al., 2013), our results demonstrate that deletion of MAP1B shifts the size distribution of growth cones toward larger size classes. In addition, the shape of MAP1B-deficient growth cones is changed, they are wider and shorter compared to wild-type growth cones of the same size (Figure S5). How could these changes be related to altered microtubule dynamics? It has been shown in HeLa cells that dynamic microtubules which polymerize all the way to the leading edge are essential for homeostatic cell length control (Picone et al., 2010). Moreover, interfering with DRG neuron microtubule dynamics by downregulation of kinesin KIF 3C or treatment with taxol leads to an increase in growth cone size (Gumy et al., 2013). MAP1B-null growth cones display an overall reduction in the density of dynamic microtubules, a slight reduction in the percentage of anterograde comets and a deficiency in dynamic microtubules at the leading edge. It is conceivable that these alterations impair homeostatic shape and size control in MAP1B-null growth cones.

The capacity of growth cones to directionally migrate, to respond to attractive and repulsive axon guidance cues and to turn at borders between permissive and non-permissive substrates all are contingent on dynamic microtubules (Dent and Gertler, 2003; Lowery and Van Vactor, 2009). MAP1B knockout growth cones are deficient in all these capacities (Bouquet et al., 2004, 2007; Del Rio et al., 2004; Stroissnigg et al., 2007; Meli et al., 2015). Our data show that most attributes of microtubule polymerization (number and density of growing microtubules, distribution within the growth cone, rate and duration of growth) are reduced upon deletion of MAP1B. This is in agreement with the assertion that MAP1B keeps microtubules in a dynamic state (Goold et al., 1999; Gonzalez-Billault et al., 2002; Tymanskyj et al., 2012). Our results suggest that reduced microtubule dynamics caused by deletion of MAP1B might contribute to the impaired growth cone performance of MAP1B-null neurons.

## Author contributions

AK designed the study, performed experiments, image processing, and statistical analysis and wrote the paper. IF performed experiments. TK and TM provided the YPF-EB3 transgenic mice prior to publication and read and improved the manuscript. FP designed the study and wrote the paper.

### Conflict of interest statement

The authors declare that the research was conducted in the absence of any commercial or financial relationships that could be construed as a potential conflict of interest.

## Supplementary material

Movie S1 Microtubule polymerization in an extending wild-type growth cone Wild-type mouse DRG neurons stably expressing YFP-EB3 **(top panel)** were imaged in 2-s intervals for 1 min long repetitive cycles. After spinning disk imaging, individual growth cones were segmented based on YFP cytoplasmic background fluorescence (**bottom panel:** growth cone boundary is outlined by dark blue, growth cone front is marked by cyan, magenta labels growth cone midline, green “x” corresponds to growth cone centroid). Subsequently, EB3-comets (indicated by red outlines and yellow ID numbers, **bottom panel**) within each growth cone were segmented and tracked individually using plusTipTracker. Scale bar: 10 μm.

Movie S2 Microtubule polymerization in an extending wild-type growth cone Wild-type mouse DRG neurons stably expressing YFP-EB3 **(top panel)** were imaged in 2-s intervals for one minute long repetitive cycles. After spinning disk imaging, individual growth cones were segmented based on YFP cytoplasmic background fluorescence (**bottom panel:** growth cone boundary is outlined by dark blue, growth cone front is marked by cyan, magenta labels growth cone midline, green “x” corresponds to growth cone centroid). Subsequently, EB3-comets (indicated by red outlines and yellow ID numbers, **bottom panel**) within each growth cone were segmented and tracked individually using plusTipTracker. Scale bar: 10 μm.

Movie S3 Continuously extending wild-type DRG growth cones Wild-type mouse DRG growth cones stably expressing EB3-YFP were imaged with a 10-min time resolution to capture growth cone displacements. In this movie, several wild-type growth cones are visible, all of which are classified as extending. The image sequence was combined from higher time resolution movies of YFP-EB3 comets, hence the 10-min time differences are indicated by jumps in the image sequence. Scale bar: 10 μm.

Figure S1 Growth cone and EB3-comet segmentation. Montages of wild-type **(A)** and MAP1B^−/−^ **(B)** DRG growth cones and EB3-YFP comets are displayed. The upper panels show the raw image sequence, the lower panels show segmentation results. Note that YFP-EB3 comets have unique identifiers, allowing tracing them throughout their lifetime and visual control of the segmentation outcome. On the segmented images, growth cone outline is marked with dark blue, light blue color indicates the growth cone front, magenta is the midline of the growth cone, and the green crosses mark growth cone centroids. The last images of each montage show a maximum time projection of the previous 11 frames. Scale bar: 5 μm.

Figure S2 Match between EB3 and SIR-actin segmentation. **(A)** In order to test the growth cone area estimation accuracy based on YFP-EB3 signal, DRG growth cones were labeled with SiR-actin, and imaged with dual-channel spinning disc microscopy. Images were independently segmented based on YFP and far-red channels. **(B–G)** Cumulative distribution functions (CDF) of growth cone size and morphological properties show a high degree of match between the independently segmented channels. Numbers on subplots indicate median property values. Significance values obtained from Kolmogorov-Smirnov tests are also displayed. ^*^, *p* < 0.05, n.s., not significant. Six hundred and seventy observations of fifteen growth cones from 2 independent experiments were included in these measurements. Scale bar: 5 μm.

Figure S3 Multivariate analyses confirm data reproducibility between and within experiments. PCA analysis based on 37 growth cone-variables [**(A)**, data points = 409 individual growth cones at single 2-s intervals] or 21 YFP-EB3 comet variables [**(C)**, data points = 9,577 individual YFP-EB3 comets at single time-points)—color-coded with respect to the mouse from which the DRGs were taken—reveal high overlap between growth cone and comet observations of independent experiments (data of wild-type mice shown.) Similar analyses of growth cone **(B)** or YFP-EB3 comet **(D)** data color-coded by measurements performed at 0, 10, and 20 min of observation time reflect temporal stability within experimental conditions.

Figure S4 Distribution of wild-type and MAP1B-null growth cones according to growth cone size. **(A)** Cumulative distribution function of growth cone area of wild-type and MAP1B-null growth cones. Red dashed lines indicate the selected maximum of growth cone size bins (110 μm^2^ for wild-type, 170 μm^2^ for MAP1B-null growth cones). Number of observations **(B)** and number of growth cones **(C)** in the different growth cone size bins are shown. The number of observations was applied in the statistical comparisons of Figures 3A–F and Figures S5A,B.

Figure S5 Growth cone morphological properties scale with increasing growth cone area. Growth cone length **(A)** and growth cone width **(B)** are both increasing as growth cone size increases. Note that wild-type growth cones are longer but less wide than MAP1B-null growth cones of the corresponding size. **(C)** Growth cone eccentricity was calculated in accordance with the MATLAB “regionprops” function as the ratio of the distance between the foci (F1 and F2) and the major axis of the ellipse fitted around the segmented growth cone. **(D)** Growth cone eccentricity decreases with the increase of growth cone, but to a higher extent in MAP1B-null growth cones than in wild-type. ^*^, *p* < 0.05, ^**^, *p* < 0.01, ^***^, *p* < 0.001, n.s., non-significant; according to Wilcoxon rank-sum test.

Figure S6 Continuous, coarse-grained, and random sampling of growth cone area. Microtubule growth rate is depicted as an example in these plots. The same sampling procedure was performed for all variables presented in Figure 3 and Figure S5. **(A)**, Scatter plot of growth cone area vs. per growth cone median comet speed in wild-type (blue) and MAP1B-null (orange) neurons. One circle corresponds to a single time point observation of an individual growth cone. Next, a sliding window sampling was performed (shown on inset). Briefly, comet speed observations were sorted according to increasing growth cone size. Growth cone area was divided into windows consisting of 300 observations. In iterative steps, this sampling window was moved towards larger growth cone areas, so that it contained 220 observations from the previous window (shown by overlapping yellow rectangles). This method ensured continuous sampling of growth cone area. **(B)** Observations within each sampling window were bootstrap sampled (100 times resampling with replacement). Medians (circles) and 95% confidence intervals (squares) of these samplings are depicted for wild-type and MAP1B^−/−^ growth cones. Re-aggregation of these bootstrapped results according to the selected growth cone area-bins provided the basis of the statistics and plots shown in Figure 3 and Figure S5. **(C)** To estimate the effect of aggregating bin size on comet speed patterns, comet speed data was grouped by growth cone area bins of 10 μm^2^ width. Note that also this binning shows the monotonic wild-type and the non-monotonic trend of the MAP1-null growth cones (Figure 3C). **p* < 0.05, ***p* < 0.01, ****p* < 0.001, n.s. = non-significant; according to Wilcoxon rank-sum test. **(D)** When comet speed observations were sorted based on a randomized order of growth cone size, the trends shown in **(B)** are not discernible. This indicates that growth cone area size impacts microtubule growth rate to a degree beyond random association.

Figure S7 Distribution of YFP-EB3 comet angles in wild-type vs. MAP1B-null growth cones of different sizes. YFP-EB3 comet angles were measured between the vector defined by the start- and endpoints of each EB3-YFP comet path and the vector defined by the growth cone basis and growth cone tip. Only comets tracked for at least 10 s were included in this analysis. Comet angles were classified as anterograde (comet moved toward growth cone front), retrograde (backward, moved away from growth cone front) or lateral (moved sideways) as follows: anterograde: 0° = < = “120°” 240° < = “comet” angle.

Figure S8 Extending and retracting growth cones mainly vary in growth cone morphology-related properties. **(A)** Principal component analysis of extending and retracting wild-type growth cones. Percentages show the fraction of total variance captured by principal component (PC). Outlined circles indicate the population centers of mass. Each dot corresponds to a multivariate observation of a growth cone at a single time point. Note the substantial overlap between the two growth cone subpopulations in the multivariate feature space. Number of growth cone observations: extending: 13553, retracting: 765. **(B)** List of top 15 growth cone and YFP-EB3 comet properties of PC1 (38% of total variance), ranked by their contribution (PCA coefficient values in PC1). **(C)**, Cumulative distribution function of growth area of extending and retracting wild-type growth cones. Dashed vertical line indicates the upper boundary (20 μm^2^) of growth cone areas included in this analysis. Data from at least 5 growth cones per growth cone size bin have been included.

Table S1 Definition of variables. This table describes the quantitative properties measured in this study on diverse spatial scales: growth cone-level (Sheet 1) and YFP-EB3 comet-level (Sheet 2). Corresponding units, detailed descriptions and calculation methods are also included.

Table S2 Growth cone and EB3-comet datasets. Available at: https://figshare.com/s/a33f8e0a30df1e0c0bcf. This table includes the quantitative multiscale data underlying this manuscript. Variables are described in Table S1. In addition to these variables Table S2 contains the coordinates of the growth cone and YFP-EB3 comet centroids. Additionally, the first 9 columns are index columns containing: 1: File name (structure: date_coating_dish_field_channel_time point_time interval_number in image sequence); 2: Growth cone ID; 3: Growth cone behavior; 4: Time point of the 1-min comet measurement. t1 = time 0, t2 = 10 min later, t3 = another 10 min later; 5: ID of ibidi dish used in the specific experiment; 6: The microscope field ID within the given dish; 7: The ID of the mouse the DRG neurons were isolated from; 8: Mouse genotype; 9: Mouse sex. These index columns match between Sheet “growth cone” and Sheet “EB3-comet.” For example, the rows belonging to Growth cone ID = 1 of Sheet 2 contain all data of the YFP-EB3 comets detected within Growth cone ID = 1. Hence, data between the different spatial scales (growth cones and comets) were connected and filtered for the calculation of growth cone level comet statistics (for example, median distance to front) according to these index columns. In the EB3-comet data sheet (Sheet 2), an additional index “Comet counter” (column 10) is added. “No data” (due to no comets present or derivative calculations of rate of change type of data) is indicated by NaN.
