# Supplementary material for: Neuronal Growth Cone Size-Dependent and -Independent Parameters of Microtubule Polymerization
Source: Front Cell Neurosci. 2018 Jul 17;12:195. doi: 10.3389/fncel.2018.00195 (PMC6056669; doi:10.3389/fncel.2018.00195)
Supplement: Figure S1 — Growth cone and EB3-comet segmentation. Montages of wild-type (A) and MAP1B−/− (B) DRG growth cones and EB3-YFP comets are displayed. The upper panels show the raw image sequence, the lower panels show segmentation results. Note that YFP-EB3 comets have unique identifiers, allowing tracing them throughout their lifetime and visual control of the segmentation outcome. On the segmented images, growth cone outline is marked with dark blue, light blue color indicates the growth cone front, magenta is the midline of the growth cone, and the green crosses mark growth cone centroids. The last images of each montage show a maximum time projection of the previous 11 frames. Scale bar: 5 μm. [file Image_1.pdf]

frontiers

Supplementary Material Images

Neuronal growth cone size-dependent and -independent parameters of microtubule polymerization

Alexa Kiss, Irmgard Fischer, Tatjana Kleele, Thomas Misgeld, and Friedrich Propst*

*** Correspondence:** Corresponding Author: friedrich.propst@univie.ac.at

Figure S1

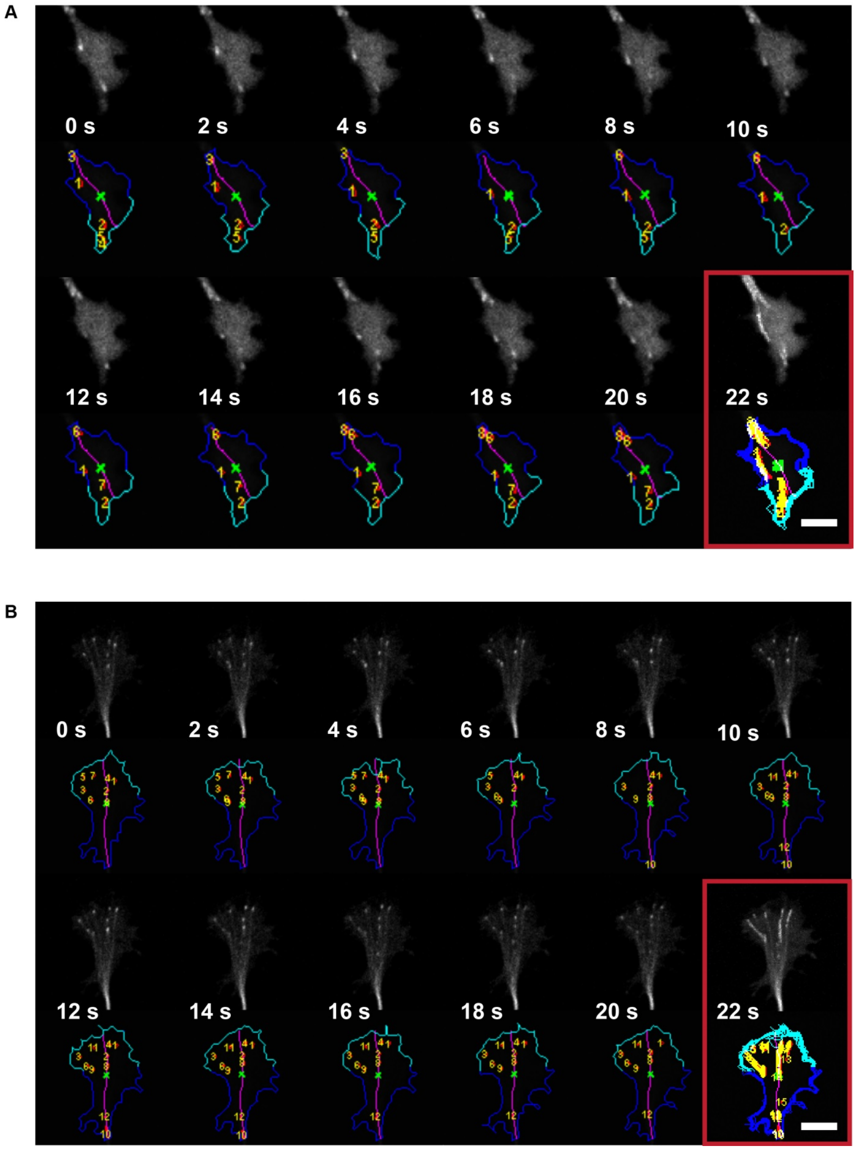

Figure S2

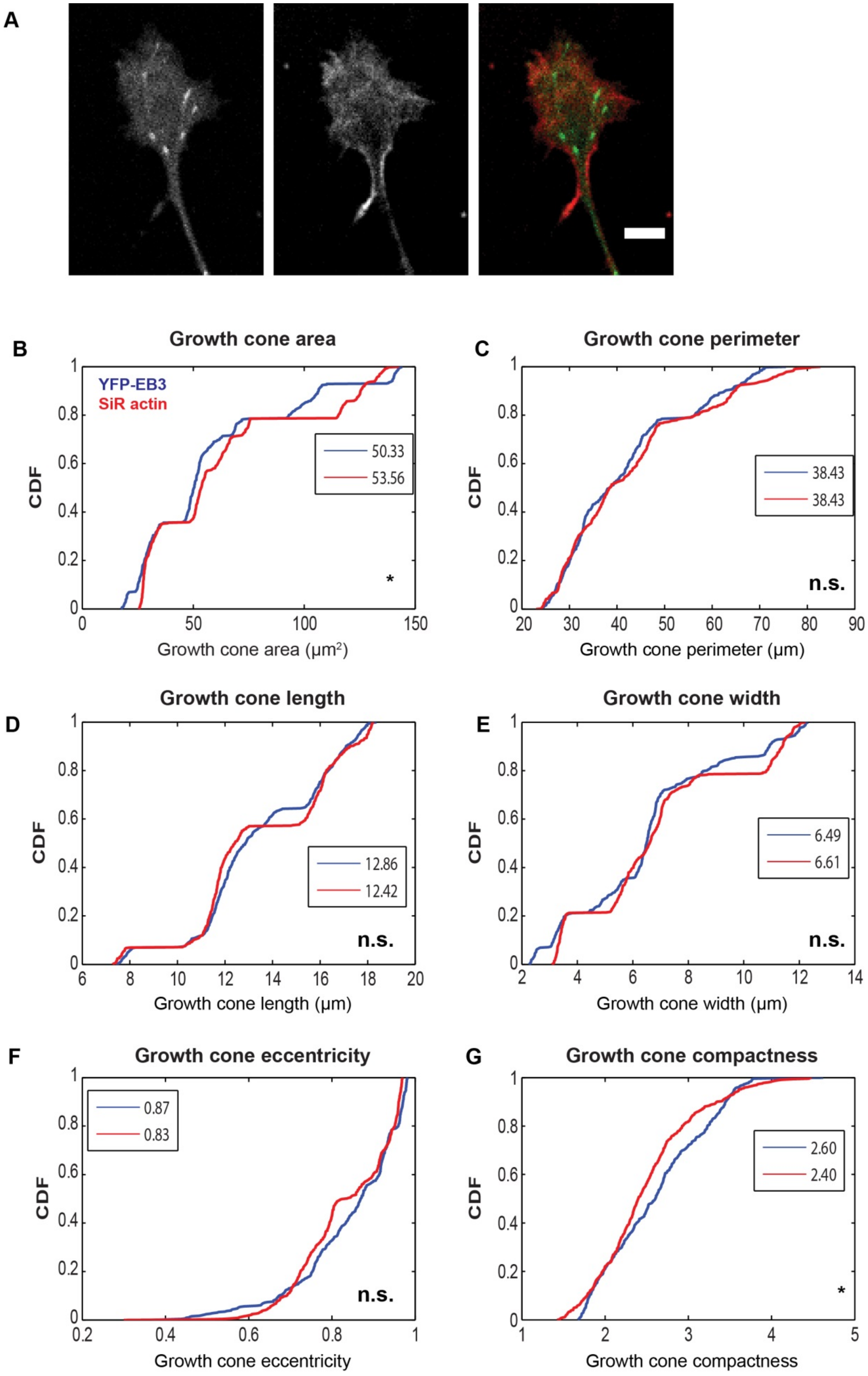
A
B
Growth cone area
YFP-EB3
SiR actin
50.33
53.56
*
CDF
Growth cone area (μm²)
C
Growth cone perimeter
38.43
38.43
n.s.
CDF
Growth cone perimeter (μm)
D
Growth cone length
12.86
12.42
n.s.
CDF
Growth cone length (μm)
E
Growth cone width
6.49
6.61
n.s.
CDF
Growth cone width (μm)
F
Growth cone eccentricity
0.87
0.83
n.s.
CDF
Growth cone eccentricity
G
Growth cone compactness
2.60
2.40
*
CDF
Growth cone compactness

Figure S3

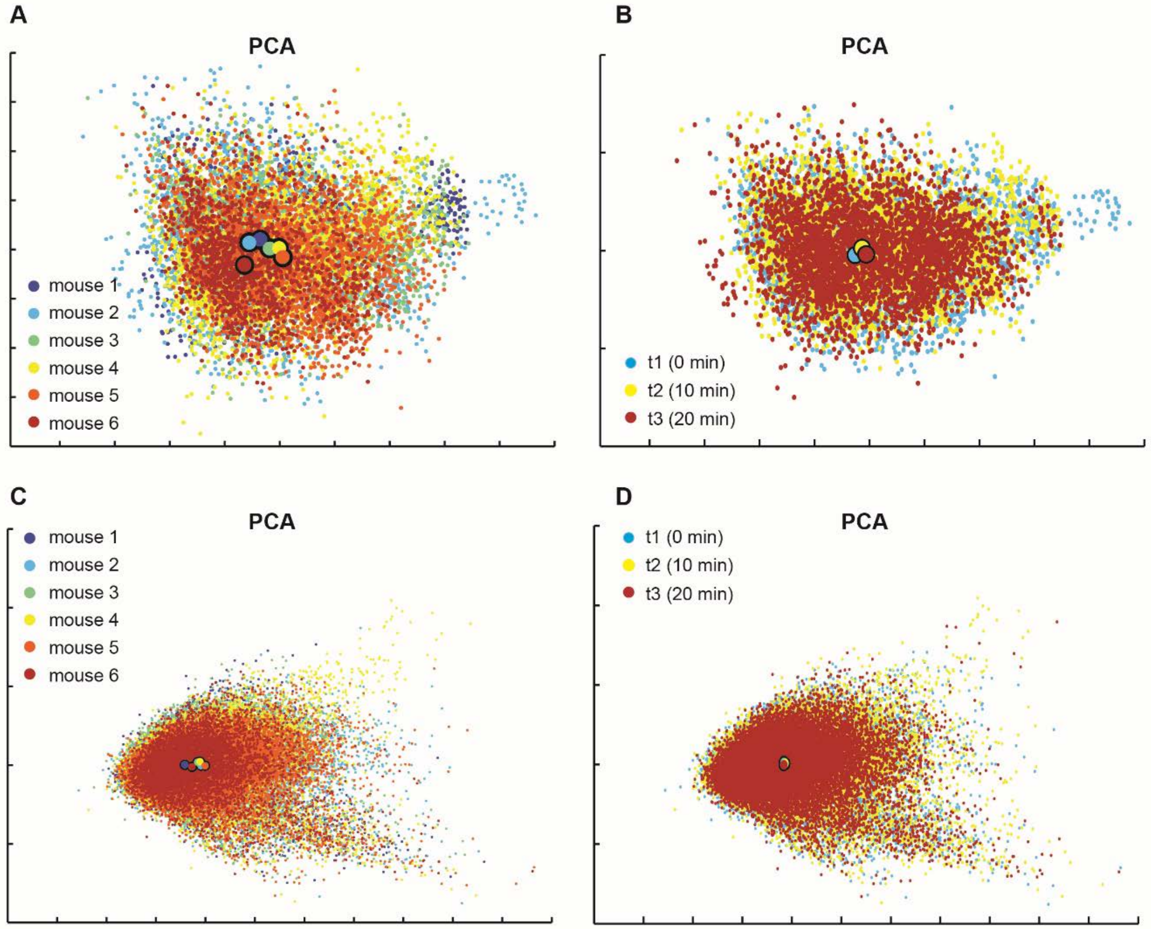

A
PCA
mouse 1
mouse 2
mouse 3
mouse 4
mouse 5
mouse 6
B
PCA
t1 (0 min)
t2 (10 min)
t3 (20 min)
C
PCA
mouse 1
mouse 2
mouse 3
mouse 4
mouse 5
mouse 6
D
PCA
t1 (0 min)
t2 (10 min)
t3 (20 min)

Figure S4

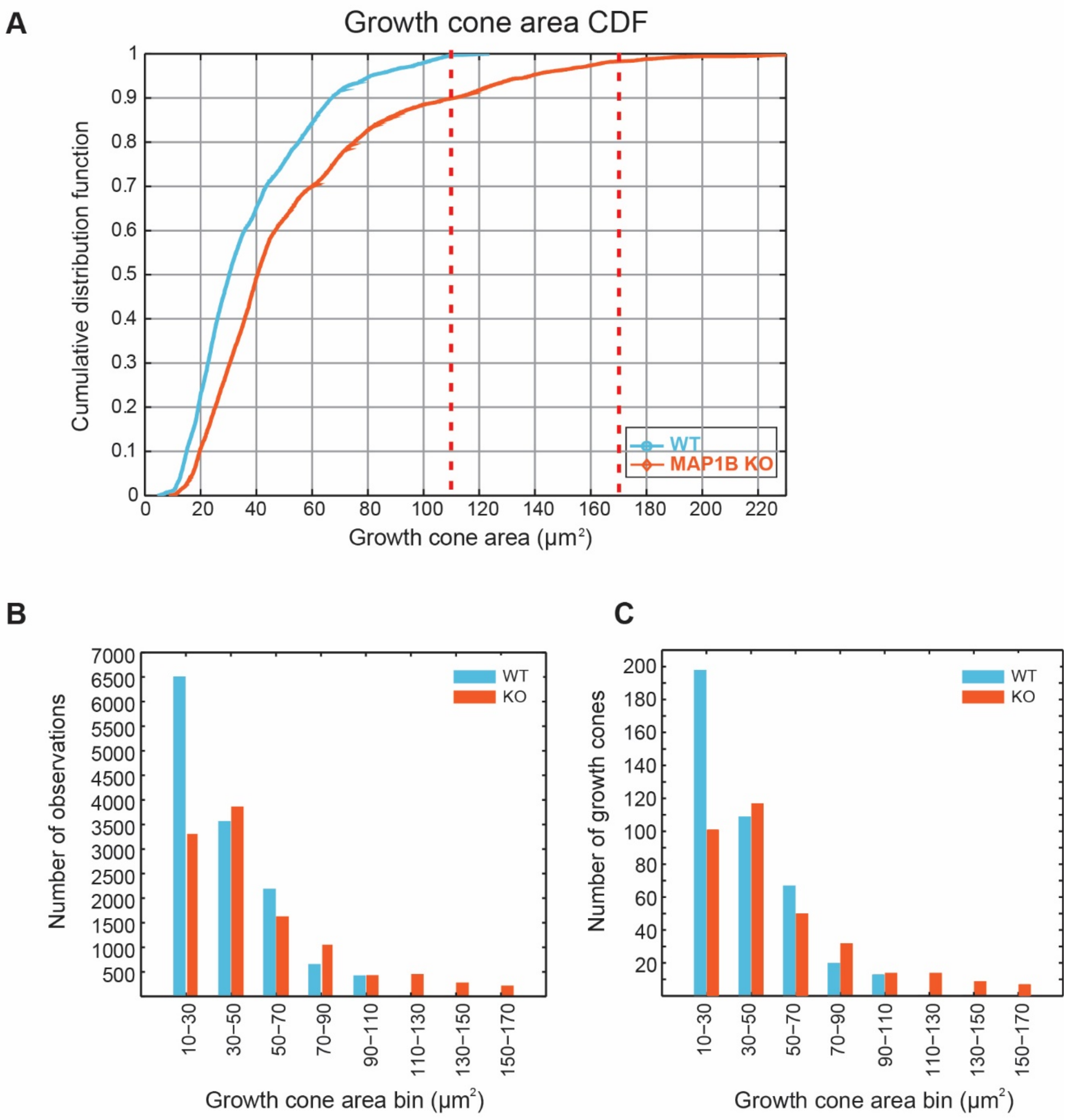
A
Growth cone area CDF
Cumulative distribution function
Growth cone area (μm²)
WT
MAP1B KO
0
0.1
0.2
0.3
0.4
0.5
0.6
0.7
0.8
0.9
1
0
20
40
60
80
100
120
140
160
180
200
220
B
Number of observations
7000
6500
6000
5500
5000
4500
4000
3500
3000
2500
2000
1500
1000
500
WT
KO
10–30
30–50
50–70
70–90
90–110
110–130
130–150
150–170
Growth cone area bin (μm²)
C
Number of growth cones
200
180
160
140
120
100
80
60
40
20
WT
KO
10–30
30–50
50–70
70–90
90–110
110–130
130–150
150–170
Growth cone area bin (μm²)

Figure S5

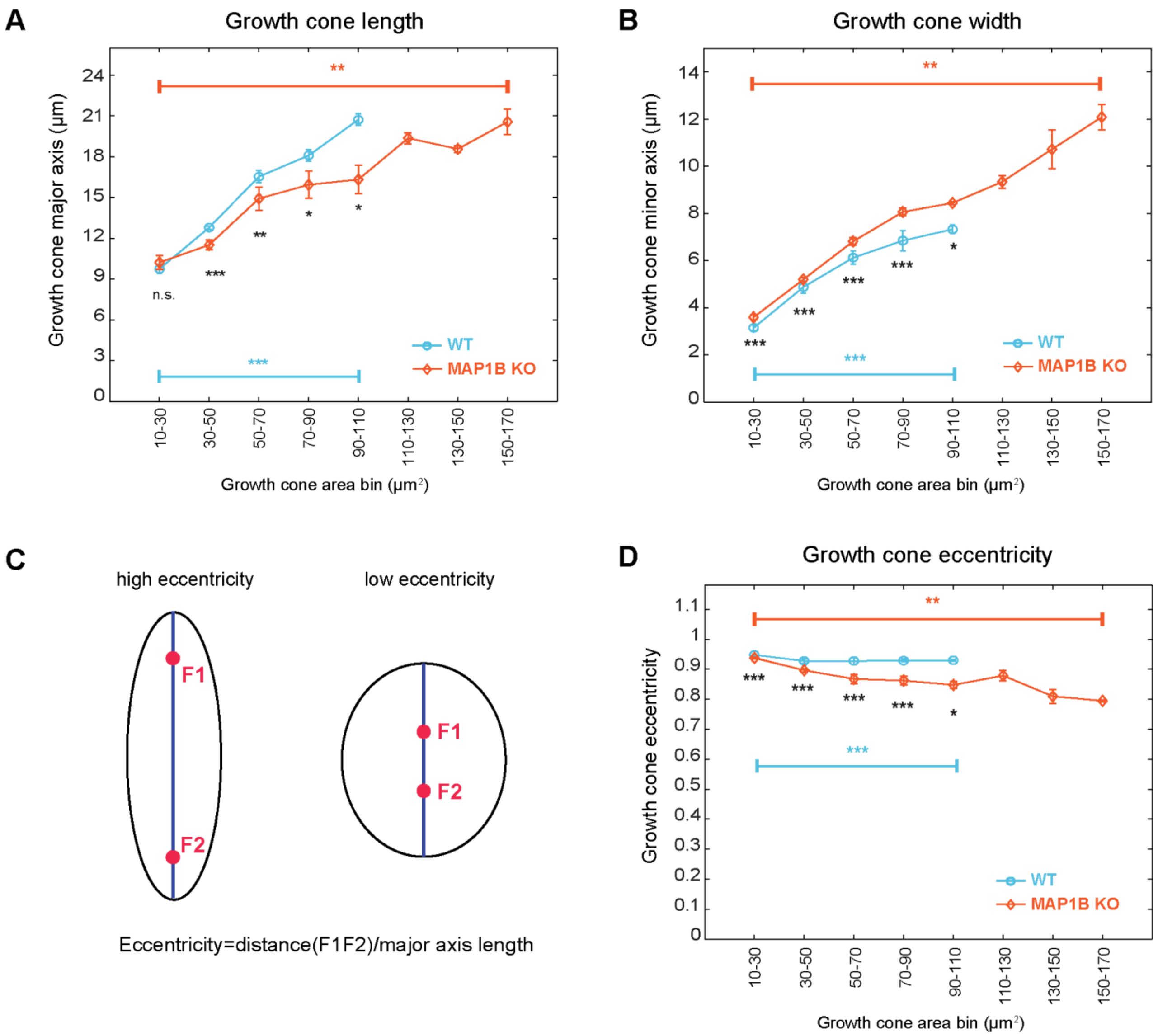

A
Growth cone length
Growth cone major axis (µm)
Growth cone area bin (µm²)
WT
MAP1B KO
n.s.
B
Growth cone width
Growth cone minor axis (µm)
Growth cone area bin (µm²)
WT
MAP1B KO
C
high eccentricity
low eccentricity
F1
F2
Eccentricity=distance(F1F2)/major axis length
D
Growth cone eccentricity
Growth cone eccentricity
Growth cone area bin (µm²)
WT
MAP1B KO

Figure S6

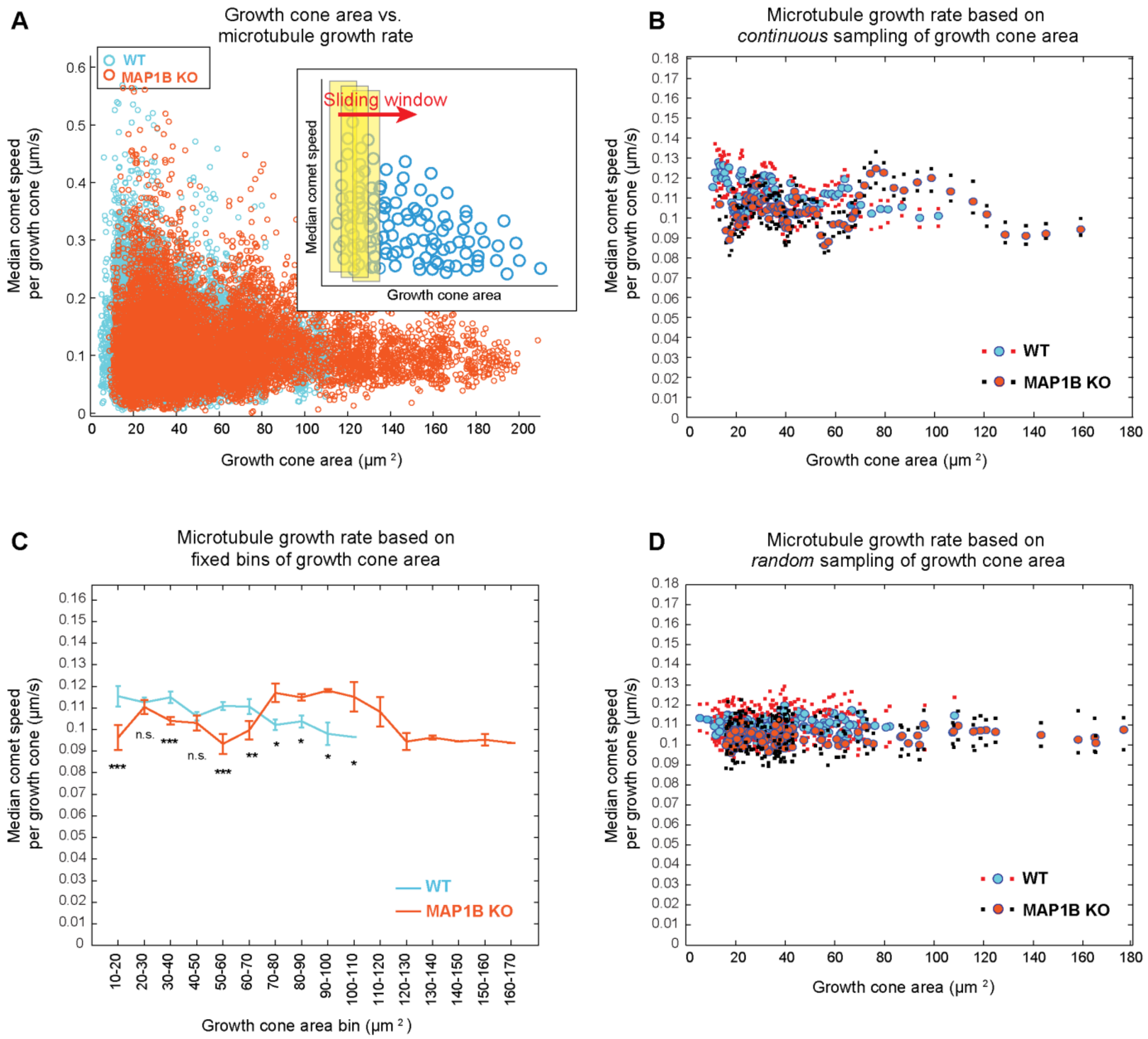
A
Growth cone area vs. microtubule growth rate
WT
MAP1B KO
Sliding window
Median comet speed
Growth cone area
Median comet speed per growth cone (µm/s)
Growth cone area (µm 2)
B
Microtubule growth rate based on *continuous* sampling of growth cone area
WT
MAP1B KO
Median comet speed per growth cone (µm/s)
Growth cone area (µm 2)
C
Microtubule growth rate based on fixed bins of growth cone area
WT
MAP1B KO
Median comet speed per growth cone (µm/s)
Growth cone area bin (µm 2)
D
Microtubule growth rate based on *random* sampling of growth cone area
WT
MAP1B KO
Median comet speed per growth cone (µm/s)
Growth cone area (µm 2)

Figure S7

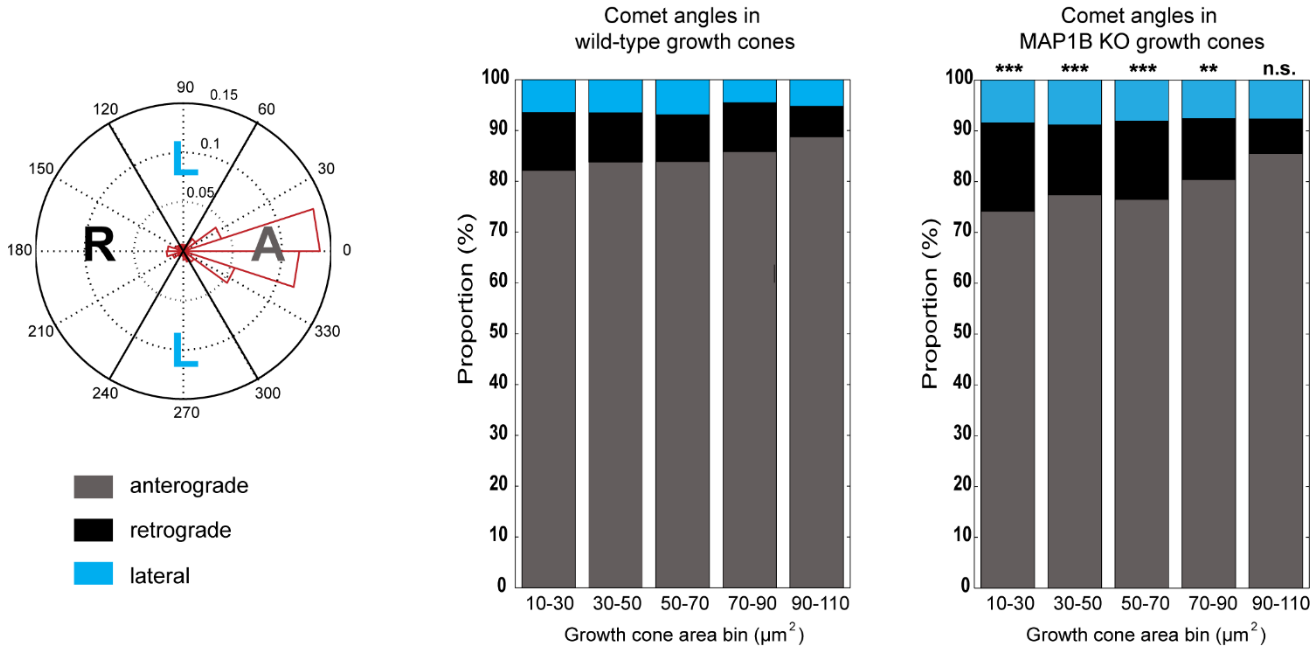

90
0.15
120
60
0.1
150
30
0.05
180
0
210
330
240
300
270
R
A
L
L
anterograde
retrograde
lateral
Comet angles in
wild-type growth cones
Proportion (%)
100
90
80
70
60
50
40
30
20
10
0
10-30
30-50
50-70
70-90
90-110
Growth cone area bin (μm²)
Comet angles in
MAP1B KO growth cones
***
***
***
**
n.s.

Figure S8

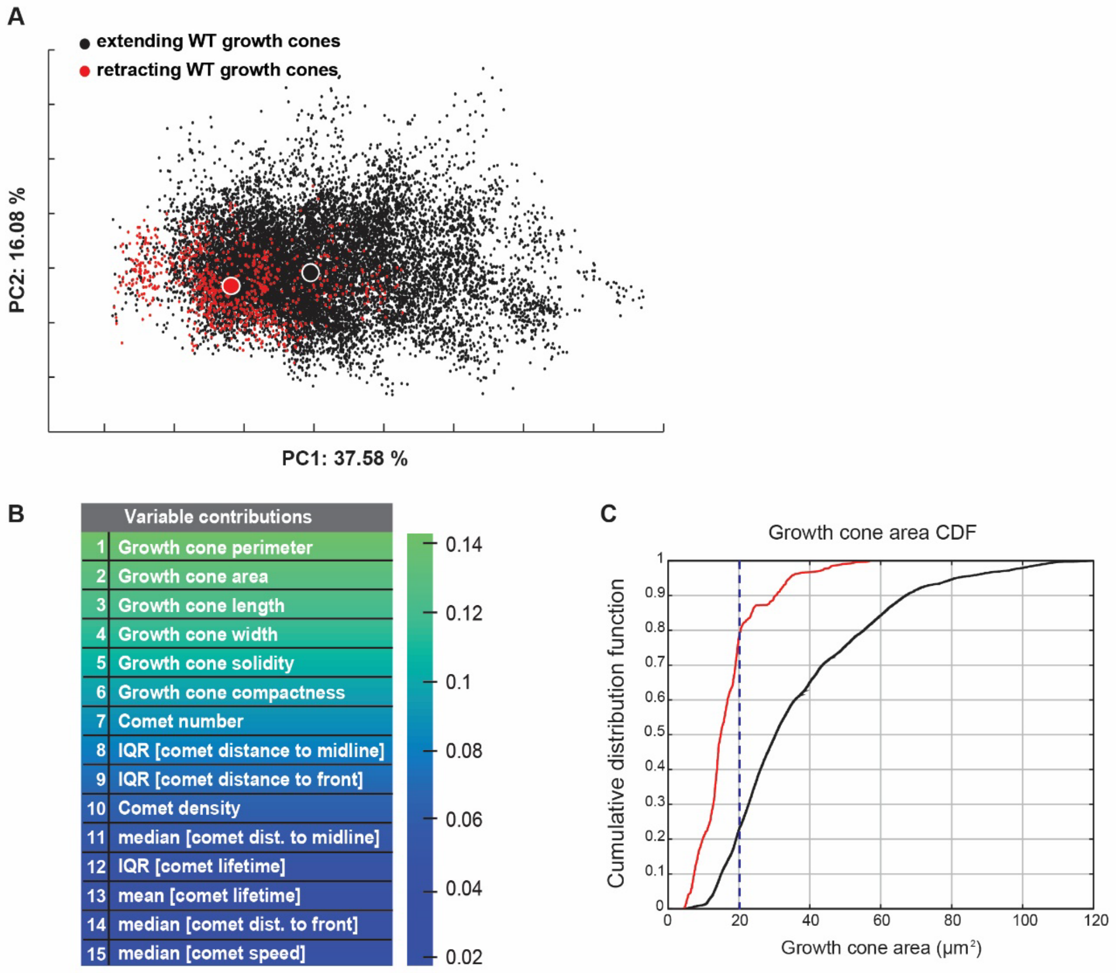
A
extending WT growth cones
retracting WT growth cones
PC2: 16.08 %
PC1: 37.58 %
B
Variable contributions
1 Growth cone perimeter
2 Growth cone area
3 Growth cone length
4 Growth cone width
5 Growth cone solidity
6 Growth cone compactness
7 Comet number
8 IQR [comet distance to midline]
9 IQR [comet distance to front]
10 Comet density
11 median [comet dist. to midline]
12 IQR [comet lifetime]
13 mean [comet lifetime]
14 median [comet dist. to front]
15 median [comet speed]
0.14
0.12
0.1
0.08
0.06
0.04
0.02
C
Growth cone area CDF
Cumulative distribution function
Growth cone area (μm²)
0
20
40
60
80
100
120
0.1
0.2
0.3
0.4
0.5
0.6
0.7
0.8
0.9
1
